# Selective dysregulation of ROCK2 activity promotes aberrant transcriptional networks in ABC diffuse large B-cell lymphoma

**DOI:** 10.1038/s41598-020-69884-1

**Published:** 2020-08-04

**Authors:** Edd Ricker, Akanksha Verma, Rossella Marullo, Sanjay Gupta, Chao Ye, Tania Pannellini, Michela Manni, Wayne Tam, Giorgio Inghirami, Olivier Elemento, Leandro Cerchietti, Alessandra B. Pernis

**Affiliations:** 1 Autoimmunity and Inflammation Program, Hospital for Special Surgery, 535 East 70th Street, New York, NY 10021 USA; 2 Graduate Program in Immunology and Microbial Pathogenesis, Weill Cornell Graduate School of Medical Sciences, New York, NY USA; 3 Department of Physiology and Biophysics, Caryl and Israel Englander Institute for Precision Medicine, Weill Cornell Medicine, New York, NY USA; 4 Hematology and Oncology Division, Weill Cornell Medicine, New York, NY USA; 5 Research Division and Precision Medicine Laboratory, Hospital for Special Surgery, New York, NY USA; 6 Department of Pathology and Laboratory Medicine, Weill Cornell Medicine, New York, NY USA; 7 David Z. Rosensweig Genomics Research Center, Hospital for Special Surgery, New York, NY USA; 8 Department of Medicine, Weill Cornell Medicine, New York, NY USA

**Keywords:** Non-hodgkin lymphoma, B cells, Kinases

## Abstract

Activated B-cell-like diffuse large B-cell lymphoma (ABC-DLBCL) is an aggressive subtype of lymphoma usually associated with inferior outcomes. ABC-DLBCL exhibits plasmablastic features and is characterized by aberrancies in the molecular networks controlled by IRF4. The signaling pathways that are dysregulated in ABC-DLBCL are, however, not fully understood. ROCK2 is a serine-threonine kinase whose role in lymphomagenesis is unknown. Here we show that ROCK2 activity is constitutively dysregulated in ABC-DLBCL but not in GCB-DLBCL and BL. We furthermore show that ROCK2 phosphorylates IRF4 and that the ROCK2-mediated phosphorylation of IRF4 modulates its ability to regulate a subset of target genes. In addition to its effects on IRF4, ROCK2 also controls the expression of MYC in ABC-DLBCL by regulating MYC protein levels. ROCK inhibition furthermore selectively decreases the proliferation and survival of ABC-DLBCL in vitro and inhibits ABC-DLBCL growth in xenograft models. Thus, dysregulated ROCK2 activity contributes to the aberrant molecular program of ABC-DLBCL via its dual ability to modulate both IRF4- and MYC-controlled gene networks and ROCK inhibition could represent an attractive therapeutic target for the treatment of ABC-DLBCL.

## Introduction

B-cell non-Hodgkin lymphoma (B-NHL) encompasses several distinct subtypes, which include Burkitt’s lymphoma (BL) and diffuse large B-cell lymphoma (DLBCL)^1,2^. While BL is derived from GC dark zone B-cells^3^, DLBCL can be subdivided into molecular subtypes based on transcriptional profiles^1,2,4^. The two major DLBCL subtypes are Germinal Center B-cell-like (GCB) DLBCL, whose transcriptome resembles that of light zone germinal center (GC) B-cells, and Activated B-cell-like (ABC) DLBCL, which instead expresses markers that normally accompany plasmablast commitment^1,2,5^. These distinctions have important clinical implications since patients with ABC DLBCL often exhibit worse survival than patients with GCB DLBCL^1,2^. Patient prognosis is also impacted by the presence of additional abnormalities, such as the expression of *MYC* and *BCL2*, which can result from either translocations or over-expression^6,7^.

One of the key features distinguishing GCB- from ABC-DLBCL is the expression of the transcription factor IRF4, which is expressed in ABC-DLBCL, but not GCB-DLBCL^8^. IRF4 is an essential regulator of multiple immune subsets, including T and B-cells^9,10^. Within the T-cell compartment, IRF4 is induced upon T-cell receptor (TCR) signaling and mediates the differentiation of several T-helper cell (T_H_) subsets, such as T_H_17 and T_FH_ cells and the production of key cytokines like IL-21, a major regulator of humoral responses^10,11^. In B-cells, IRF4 is upregulated upon B-cell activation and regulates multiple stages of B-cell differentiation, including GC B-cell formation, class switch recombination, GC exit, and plasma cell (PC) differentiation^9,12–14^. The multifaceted actions of IRF4 rely on its ability to interact with multiple partners including ETS-, AP1-, and other IRF- family members and target distinct regulatory elements^13–17^. Notably, dysregulation of IRF4 expression and/or activity promotes oncogenesis in several B-cell malignancies, including ABC-DLBCL and Multiple Myeloma (MM)^8,18^. The diverse tumorigenic effects of IRF4 have been associated with context-dependent functions of IRF4, whereby its interaction with the ETS-family member SPI-B is critical for the survival of ABC-DLBCL, while a positive transcriptional auto-regulatory loop between IRF4 and MYC fuels myeloma cell survival^8,18^.

Given the essential and complex roles of IRF4 in controlling immune responses, regulatory mechanisms must exist to ensure the proper execution of distinct IRF4-mediated transcriptional programs. Previous work identified the serine-threonine kinase ROCK2 as a regulator of IRF4 activity in T_H_17 cells^19^. ROCK2 and its only other family member, ROCK1, are highly homologous serine-threonine kinases that serve as major downstream effectors of the Rho subfamily of small GTPases, which includes RhoA^20,21^. Like other small GTPases, RhoA cycles between an inactive and an active state, a process controlled by Rho-guanine-nucleotide exchange factors (GEFs)^22^. Upon RhoA binding, the ROCKs undergo a conformational change resulting in kinase activation^20,21^. Genetic alterations affecting the RhoA-ROCK pathway have recently been implicated in T-cell lymphomagenesis with 60–70% of Angioimmunoblastic T-cell lymphomas (AITLs) expressing an inactivating mutation in RhoA^23–25^. Mutations in RhoA or in its upstream activators have also been identified in BL and in GCB-DLBCL^26–28^. The role of key downstream RhoA effectors, like the ROCKs, in lymphomagenesis is, however, unknown.

In this study we show that ROCK2 is constitutively activated in ABC-DLBCL but not in GCB-DLBCL or in BL. ROCK2 furthermore constitutively phosphorylates IRF4 in ABC DLBCL and modulates the IRF4-regulated transcriptional program. Silencing of ROCK2 alters the transcriptional profile of ABC-DLBCL not only by modulating IRF4 activity, but also by diminishing MYC protein levels. In line with these findings, ROCK inhibitors selectively decrease the survival of ABC-DLBCL. Thus, dysregulated ROCK2 activity can promote lymphomagenesis in ABC-DLBCL via effects on both IRF4 and MYC and inhibiting this kinase could represent a novel therapeutic approach for the treatment of ABC-DLBCL.

## Results

### IRF4 is constitutively phosphorylated in ABC-DLBCL

**Figure 1:**
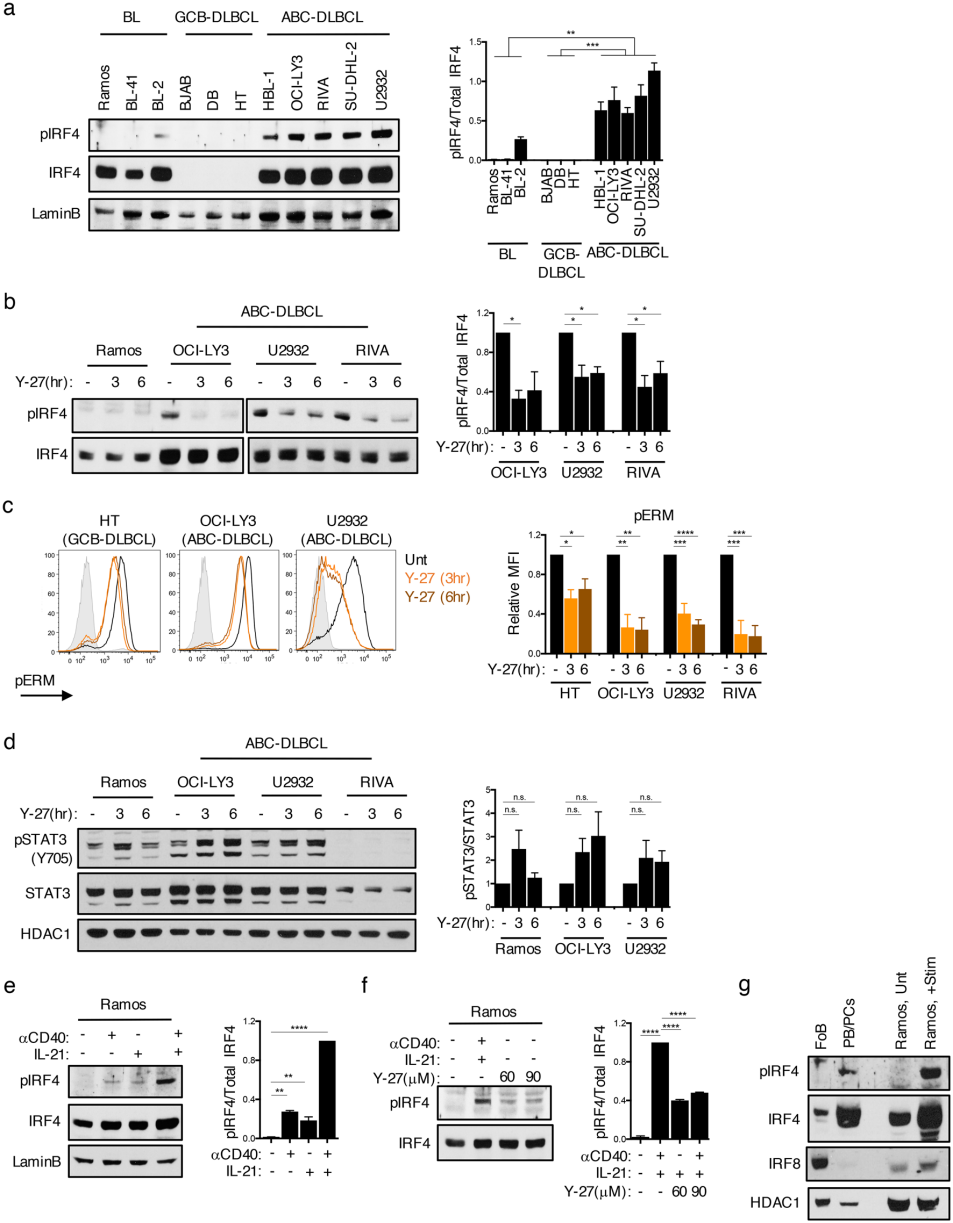
IRF4 is constitutively phosphorylated in ABC-DLBCL. (**a**) Representative immunoblot and quantifications of phosphorylated IRF4 at S446/S447 (pIRF4), total IRF4, and LaminB from nuclear extracts of BL (Ramos, BL-41, BL-2), GCB-DLBCL (BJAB, DB, HT), and ABC-DLBCL (HBL-1, OCI-LY3, RIVA, SU-DHL-2, U2932) cells. Quantifications are calculated as the densitometry ratio between pIRF4 to total IRF4 (mean ± SEM; *n* =  > 3 per cell line; *p* value by 1-way ANOVA followed by Tukey’s multiple comparisons test). (**b**) Representative immunoblot and quantifications of indicated proteins from nuclear extracts of cells either left untreated or cultured in the presence of 90 μM Y-27632 (Y-27), a pan-ROCK inhibitor. Blot separation indicates different exposures of the same blot. Quantifications are calculated as in (**a**) (mean ± SEM; *n* =  > 2 per cell line; *p* value by 1-way ANOVA followed by Dunnett’s multiple comparisons test). (**c**) Representative histograms and quantifications of phosphorylated ERM (pERM) expression in DLBCL cells either left untreated or following treatment with 90 μM Y-27 (mean ± SEM; *n* =  > 3; *p* value by 1-way ANOVA followed by Dunnett’s multiple comparisons test). (**d**) Representative immunoblot and quantifications of phosphorylated STAT3 (pSTAT3; Y705), total STAT3, and HDAC1 from nuclear extracts of cell lines either left untreated or cultured with Y-27 as in (**b**). Quantifications are calculated as the densitometry ratio of pSTAT3 to total STAT3 (mean ± SEM; *n* = 2; *p* value by 1-way ANOVA followed by Dunnett’s multiple comparisons test). (**e**) Representative immunoblot and quantifications of indicated proteins from nuclear extracts of Ramos cells treated for 6 h with various combinations of αCD40 and IL-21. Quantification is calculated as in (**a**) (mean ± SEM; *n* = 2; *p* value by 1-way ANOVA followed by Dunnett’s multiple comparisons test). (**f**) Representative immunoblot and quantifications of indicated proteins from nuclear extracts of Ramos cells pre-treated for 2 h with Y-27 before stimulation as in (**e**). Quantification is calculated as in (**e**) (mean ± SEM; *n* = 2; *p* value by 1-way ANOVA followed by Tukey’s multiple comparisons test). (**g**) Representative immunoblot of indicated proteins from lysates of sorted follicular B-cells (FoBs; Blimp1-yfp^−^CD138^-^B220^+^CD23^+^) or plasmablasts/plasma cells (PB/PCs; Blimp1-yfp^+^CD138^+^) from Blimp1-yfp reporter mice at d7 post-immunization with 100 μg NP-CGG. Ramos cells were used as a control. Data representative of 3 independent experiments. * p < 0.05, ** p < 0.01, *** p < 0.001, **** p < 0.0001.

Given the essential role of IRF4 in ABC-DLBCL and the ability of ROCK2 to phosphorylate IRF4 in T-cells^19^, we investigated whether IRF4 is aberrantly phosphorylated in ABC-DLBCL by using an antibody that detects the phosphorylation of IRF4 at S446/S447, the ROCK2 phosphorylated residues^19^. Probing of nuclear extracts demonstrated that IRF4 is highly phosphorylated in several ABC-DLBCL cell lines (Fig. 1a). In contrast, BL lines expressed no or low levels of phosphorylated IRF4, despite expressing similar levels of total IRF4 protein (Fig. 1a). Consistent with previous reports, IRF4 was not expressed in GCB-DLBCL^5,8^ (Fig. 1a). Culturing ABC-DLBCL cells in the presence of Y-27632, an inhibitor of both ROCK1 and ROCK2, decreased IRF4 phosphorylation suggesting that IRF4 phosphorylation was dependent on ROCK activity (Fig. 1b). As predicted, treatment with Y-27632 also decreased the phosphorylation of the ezrin/radixin/moesin (ERM) proteins, classic targets for both ROCK1 and ROCK2, in all DLBCL cell lines examined (Fig. 1c). As reported, ABC-DLBCL cells displayed a wide-range of constitutively phosphorylated STAT3^29^ (Fig. S1A). STAT3 phosphorylation did not correlate with the levels of phosphorylated IRF4 and, in contrast to IRF4 phosphorylation, was not diminished by Y-27632 treatment (Fig. 1d). Thus, IRF4 is constitutively phosphorylated in ABC-DLBCL in a ROCK-dependent manner.

Since ABC-DLBCLs correspond to B-cells arrested in a plasmablast (PB)-like state^1,2^, we next assessed whether T_FH_-derived signals that normally drive the differentiation of GC B-cells into plasmablast/plasma cells (PB/PCs), such as CD40 engagement and IL-21, could induce IRF4 phosphorylation. To address this possibility, we cultured Ramos cells, a human BL cell line previously employed as a model system to study GC exit^30^, in the presence of αCD40 and IL-21. Stimulation with either αCD40 or IL-21 alone led to a modest induction of IRF4 phosphorylation, while the combination of these two signals resulted in a robust increase in IRF4 phosphorylation (Fig. 1e). IRF4 phosphorylation upon αCD40 and IL-21 stimulation was inhibited by Y-27632 suggesting that it was dependent on ROCK activity (Fig. 1f). We next evaluated whether IRF4 phosphorylation could also be observed in primary PB/PCs. To this end, we immunized Blimp1-YFP mice, in which Blimp1-expressing cells can be identified via the expression of *yfp*, and sorted either follicular B-cells (B220^+^Blimp1^−^CD23^+^) or PB/PCs (Blimp1^+^CD138^+^) from the spleens of these mice. Phosphorylation of IRF4 could be detected in Blimp1^+^ PB/PCs but not in follicular B-cells (Fig. 1g), suggesting that IRF4 phosphorylation occurs in primary PB/PCs.

### ROCK2 activity is dysregulated in ABC-DLBCL

**Figure 2:**
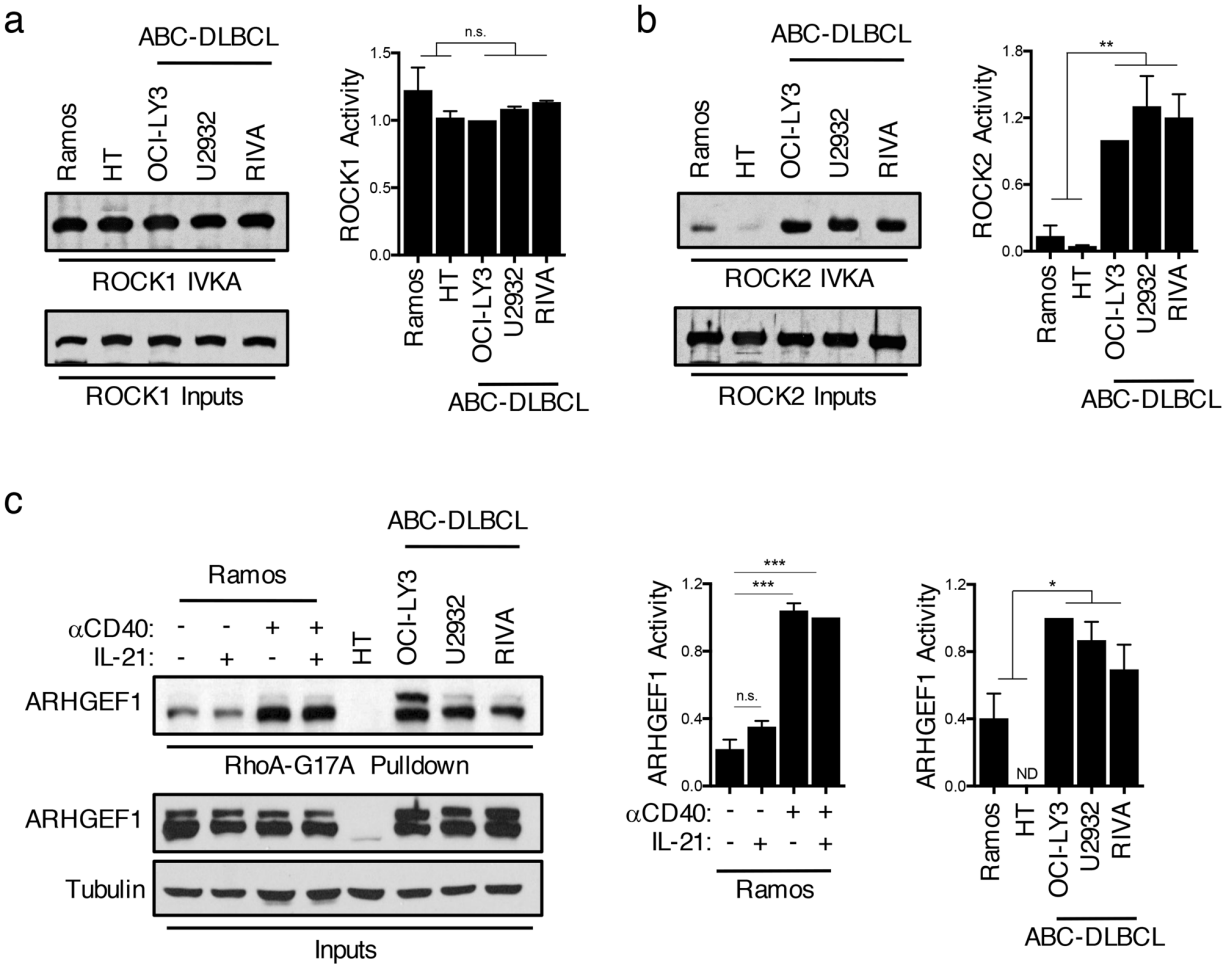
Dysregulated activation of ROCK2 in ABC-DLBCLs. (**a–b**) ROCK1 and ROCK2 kinase activity was assayed by incubating immunoprecipitated ROCK1 (**a**) or ROCK2 (**b**) from nuclear extracts of BL, GCB-DLBCL, or ABC-DLBCL cell lines with purified recombinant MYPT1 as a substrate in the presence of ATP. Phosphorylated MYPT1 (pMYPT1) was detected using an antibody against pMYPT1. Total ROCK1 or ROCK2 input levels for each sample are shown in the lower panels. Quantifications are calculated as the densitometry ratio between pMYPT1 to total ROCK input protein (mean ± SEM; *n* = 2; *p* value by unpaired two-tailed *t* test). (**c**) RhoA-G17A-conjugated agarose beads were used to pull-down active ARHGEF1 from lysates of GCB-DLBCL, ABC-DLBCL, or Ramos cells following 6 h treatment with various combinations of αCD40 and IL-21. Quantifications are calculated as the densitometry ratio between ARHGEF1 from the RhoA-G17A pull-down to ARHGEF1 input levels [mean ± SEM; *n* =  > 2; *p* value by 1-way ANOVA followed by Dunnett’s multiple comparisons test (left) or by unpaired two-tailed *t* test (right)]. * p < 0.05, ** p < 0.01, *** p < 0.001, **** p < 0.0001.

Since the phosphorylation of IRF4 detected in ABC-DLBCL was decreased by Y-27632, we directly investigated whether ROCK activation was differentially regulated in distinct subtypes of B-cell lymphomas. In order to assess ROCK1- and ROCK2-specific activation, we performed in vitro kinase assays (IVKAs), in which ROCK1 or ROCK2 was immunoprecipitated from nuclear extracts and assessed for their ability to phosphorylate recombinant MYPT1, a well-known ROCK substrate^19,31^. In contrast to ROCK1, which was activated to a similar extent in the B-NHL cell lines examined (Fig. 2a), high levels of ROCK2 activity were only observed in ABC-DLBCL lines (Fig. 2b), suggesting that ROCK2 activity is selectively dysregulated in ABC-DLBCL.

ROCK activation is primarily induced upon binding of activated RhoA, which, in turn, can be regulated by GEFs^20^. While at least 24 GEFs have been reported to activate RhoA, ARHGEF1 is known to be an important GEF regulating RhoA activity in B-cells^26,32–34^. Furthermore, we previously observed an association between ARHGEF1 activation and ROCK2 activity in CD4^+^ T-cells^31^. To assess the activation state of ARHGEF1 in B-NHL cells, we employed a RhoA-G17A pull-down assay (Fig. 2c). The RhoA-G17A mutant stably binds to active Rho-GEFs, facilitating the detection of specific GEFs that are activated under different stimulatory conditions. ARHGEF1 was highly activated in several ABC-DLBCL cell lines as well as in Ramos cells stimulated with αCD40 and IL-21 (Fig. 2c), suggesting that activation of ARHGEF1 may contribute to the activation of ROCK2 in ABC-DLBCL.

### ROCK2 regulates the expression of a subset of IRF4 target genes in stimulated B-cells

**Figure 3:**
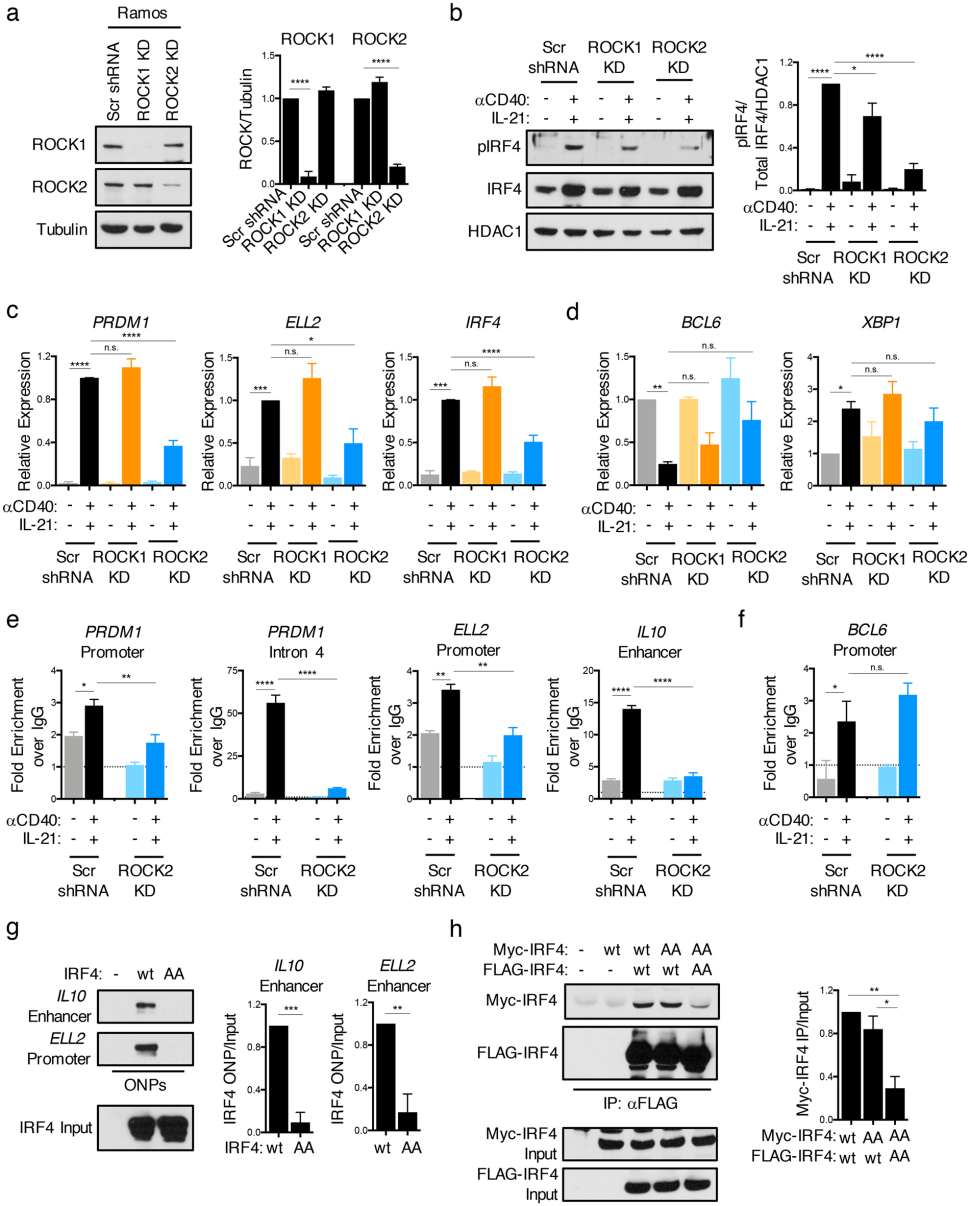
ROCK2 regulates the expression of a subset of IRF4 target genes in stimulated B-cells. (**a**) Representative immunoblot and quantifications of ROCK1 and ROCK2 from lysates of Ramos cells after stable lentiviral infection with shRNA constructs targeting either ROCK1 (ROCK1 KD), ROCK2 (ROCK2 KD), or with a scrambled shRNA control (Scr shRNA). Quantifications are calculated as the densitometry ratio between each ROCK protein to β-Tubulin (mean ± SEM; *n* = 3; *p* value by 1-way ANOVA followed by Dunnett’s multiple comparisons test). (**b–f**) Stable Ramos ROCK1 KD (orange), ROCK2 KD (blue), and Scr (black) control cells were left untreated or stimulated for 6 h with αCD40 and IL-21. (**b**) Representative immunoblot and quantifications of pIRF4 and total IRF4 from nuclear extracts of stable Ramos ROCK KD cells. Quantifications are calculated as the densitometry ratio between pIRF4 to the ratio of total IRF4 to HDAC1 (mean ± SEM; *n* = 3; *p* value by 1-way ANOVA followed by Dunnett’s multiple comparisons test). (**c–d**) Pooled RT-qPCR analysis of indicated transcripts (mean ± SEM; *n* = 4; *p* value by 1-way ANOVA followed by Dunnett’s multiple comparisons test). (**e–f**) Representative ChIP-qPCR analysis of IRF4 binding to regulatory regions in the *PRDM1*, *ELL2, IL10*, and *BCL6* loci (mean ± SD; *n* = 2; *p* value by 1-way ANOVA followed by Dunnett’s multiple comparisons test). (**g**) Oligonucleotide precipitation assays (ONPs) of extracts from 293 T cells transfected with wt or phosphomutant (AA) IRF4, assessed with biotinylated oligonucleotides from the *IL10* enhancer or the *ELL2* promoter region, followed by immunoblot of precipitated IRF4. Quantifications are calculated as the densitometry ratio between IRF4 precipitated during the ONP to input IRF4 levels (mean ± SEM; *n* = 3; *p* value by unpaired *t* test). (**h**) 293 T cells were co-transfected with MYC-tagged IRF4-wt or MYC-tagged IRF4-AA and either FLAG-tagged IRF4-wt or FLAG-tagged IRF4-AA as indicated. Immunoprecipitations were performed using an anti-FLAG antibody and analyzed by immunoblotting. Quantifications are calculated as the densitometry ratio between precipitated MYC-tagged IRF4 protein to input MYC-tagged IRF4 (mean ± SEM; *n* = 3; *p* value by 1-way ANOVA followed by Tukey’s multiple comparisons test). * p < 0.05, ** p < 0.01, *** p < 0.001, **** p < 0.0001.

The association between the activation state of ROCK2 and IRF4 phosphorylation detected in ABC-DLBCL cell lines led us to hypothesize that IRF4 phosphorylation may be primarily mediated by ROCK2. To start addressing this possibility, we first silenced ROCK1 or ROCK2 in Ramos cells with shRNA constructs targeting either of the two ROCKs (Figs. 3a, S2A). While silencing of ROCK1 had minimal effects on IRF4 phosphorylation, silencing of ROCK2 markedly decreased the phosphorylation of IRF4 upon stimulation with αCD40 and IL-21 (Fig. 3b). Thus, ROCK2 is the key ROCK isoform mediating IRF4 phosphorylation downstream of CD40 and IL-21R.

CD40 stimulation induces Ramos cells to acquire a gene expression signature resembling that of B-cells exiting the GC^30^. This transition is characterized by the IRF4-dependent downregulation of *BCL6* and the upregulation of other targets including *PRDM1*, a central hub in the PB/PC transcriptional network^30^. To assess whether IRF4 phosphorylation might affect this transition, we stimulated Ramos cells silenced for either ROCK1 (ROCK1 knockdown) or ROCK2 (ROCK2 knockdown) and monitored the expression of known IRF4 targets. The upregulation of several IRF4 target genes that play critical roles in PB/PC differentiation, including *PRDM1, ELL2*, and *IRF4* was significantly diminished in stimulated Ramos ROCK2 knockdown cells but not in ROCK1 knockdown cells (Fig. 3c). The effects of the ROCK2 knockdown on *PRDM1* and *IRF4* mRNA expression furthermore corresponded to a marked reduction in protein levels following stimulation (Fig. S2B-C). Interestingly, ROCK2 silencing did not exert global effects on IRF4 targets or key regulators of B-cell differentiation, as the expression of *BCL6* and *XBP1* was only minimally affected in ROCK2 knockdown cells (Fig. 3d). Thus, ROCK2 regulates the expression of a specific subset of IRF4 target genes in GC-like B-cells stimulated with signals that promote GC B-cell exit and PB/PC differentiation.

To assess whether ROCK2 silencing impacted the ability of IRF4 to target specific regulatory regions, we performed chromatin immunoprecipitation (ChIP) assays followed by qPCR using primers encompassing regulatory sites known to be bound by IRF4^8,18,30^. Silencing of ROCK2 resulted in a significant decrease in the binding of IRF4 to regulatory regions within the *PRDM1* locus, the *ELL2* promoter, and the *IL10* enhancer (Fig. 3e). IRF4 binding to the *BCL6* promoter was instead not consistently affected by ROCK2 knockdown as compared to the other sites examined (Fig. 3f). To further examine the effects of phosphorylation on the DNA binding activity of IRF4, we transfected 293 T cells, which contain baseline ROCK activity, with either a wild-type IRF4 expression construct (IRF4-wt) or a construct encoding a mutant of IRF4 that cannot undergo S446/S447 phosphorylation (IRF4-AA) and performed oligonucleotide precipitation assays (ONPs) using oligonucleotides containing the IRF4 binding sites in the *IL10* enhancer or the *ELL2* promoter (Fig. 3g). Binding of the IRF4-AA mutant protein to both sites was markedly diminished as compared to the binding observed with IRF4-wt protein. To assess whether IRF4 phosphorylation by ROCK2 could alter its ability to homodimerize, we furthermore transfected 293 T cells with various combinations of differentially tagged IRF4-wt and IRF4-AA expression constructs followed by co-immunoprecipitations. IRF4 homodimers could be observed when FLAG-IRF4-wt and MYC-IRF4-wt proteins were co-expressed or when FLAG-IRF4-wt was expressed with MYC-IRF4-AA, but not when FLAG-IRF4-AA and MYC-IRF4-AA were co-expressed (Fig. 3h). These data suggest that the ROCK2-dependent phosphorylation of IRF4 promotes IRF4 homodimerization and regulates its binding to selected regulatory regions.

### ROCK2 regulates the expression of IRF4-repressed targets in ABC-DLBCL

**Figure 4:**
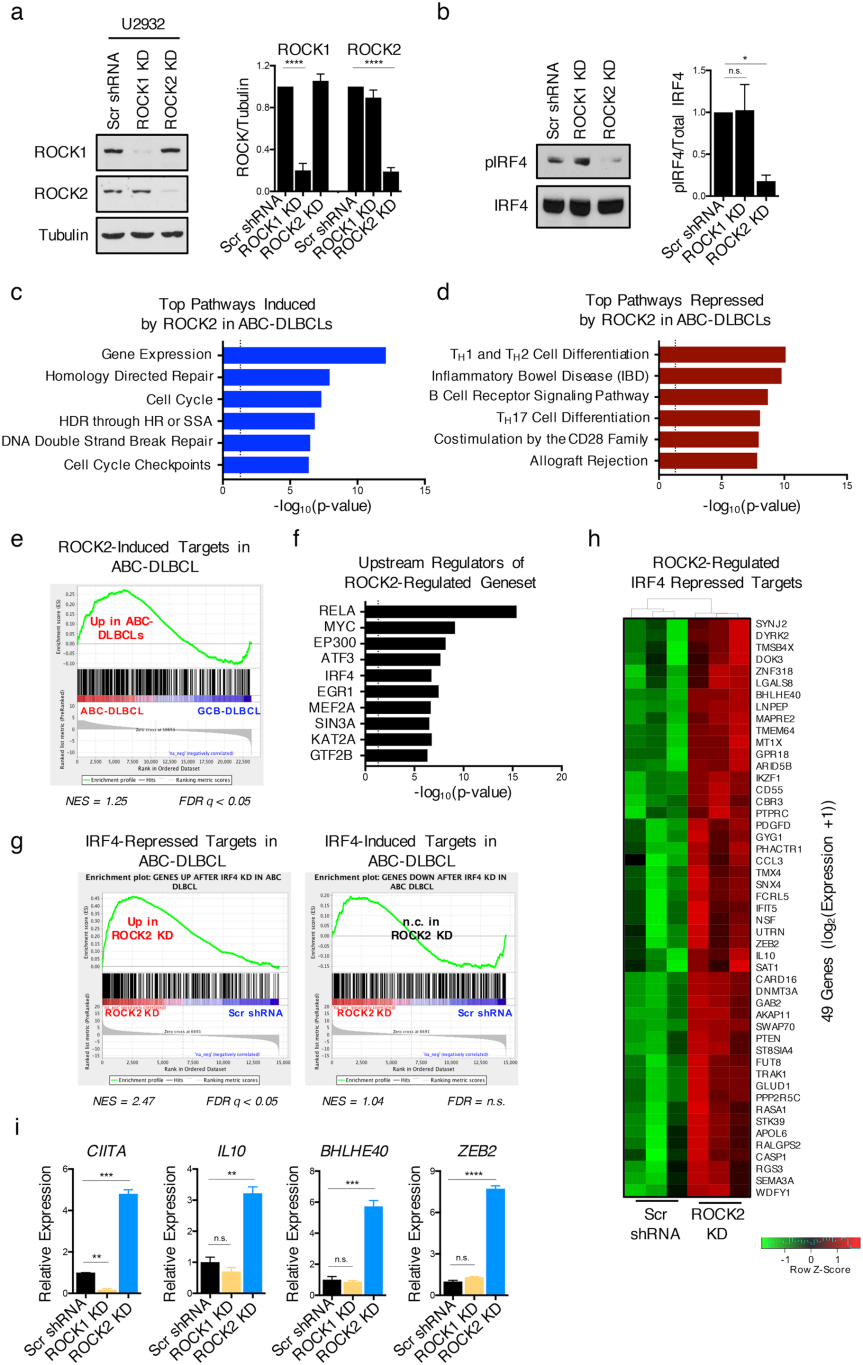
ROCK2 regulates the expression of IRF4-repressed targets in ABC-DLBCL. (**a**) Representative immunoblot and quantifications of ROCK1 and ROCK2 from lysates of U2932 cells after stable lentiviral infection with shRNA expression constructs targeting either ROCK1 (ROCK1 KD), ROCK2 (ROCK2 KD) or with a scrambled shRNA control (Scr shRNA). Quantifications are calculated as the densitometry ratio between each ROCK protein to β-Tubulin (mean ± SEM; *n* = 4; *p* value by 1-way ANOVA followed by Dunnett’s multiple comparisons test). (**b**) Representative immunoblot and quantifications of phosphorylated IRF4 at S446/S447 (pIRF4) and total IRF4 from nuclear extracts of stable U2932 ROCK KD cells. Quantifications are calculated as the densitometry ratio between pIRF4 to total IRF4 (mean ± SEM; *n* = 5; *p* value by 1-way ANOVA followed by Dunnett’s multiple comparisons test). (**c–g**) RNA-seq analysis was performed on U2932 ROCK2 KD cells. (**c–d**) Plots showing the -log_10_ (*p* value) values for the top over-represented pathways from the genes induced (**c**) or repressed (**d**) by ROCK2 in U2932. Dotted lines indicate significance cutoffs at *p* = 0.05. (**e**) GSEA plot showing the significant enrichment of the ROCK2-induced geneset from U2932 (Table S1) in primary ABC-DLBCL cases. (**f**) Plot showing the top enriched upstream regulators of the ROCK2-regulated geneset in U2932 using EnrichR analysis. Dotted line indicates significance cutoff at *p* = 0.05. (**g**) GSEA plot showing the significant enrichment of previously defined IRF4-repressed and IRF4-induced genesets from ABC-DLBCLs in U2932 ROCK2 KD cells. (**h**) Heat map depiction of target genes identified in (**f**) that are contributing to the enrichment of the “IRF4-repressed targets in ABC-DLBCL” pathway and are differentially expressed in U2932 ROCK2 KD cells. (**i**) Representative RT-qPCR analysis of the indicated genes in U2932 ROCK1 KD *(orange)*, ROCK2 KD *(blue)*, or scrambled shRNA control cells *(black)*. Data representative of 3 independent experiments (mean ± SD; *p* value by 1-way ANOVA followed by Dunnett’s multiple comparisons test). * p < 0.05, ** p < 0.01, *** p < 0.001, **** p < 0.0001.

The ability of ROCK2 to regulate the activity of IRF4 raised the possibility that dysregulated ROCK2 activation in ABC-DLBCL could contribute to their phenotype. To directly evaluate this possibility, we silenced ROCK2 in U2932 cells, generating U2932 ROCK2 knockdown cells (Figs. 4a, S3A). U2932 ROCK1 knockdown cells were also generated to directly compare the roles of the two ROCKs in these cells (Figs. 4a, S3A). In line with the results obtained in stimulated Ramos cells, the constitutive phosphorylation of IRF4 in U2932 cells was prominently decreased upon silencing of ROCK2 but not ROCK1 (Fig. 4b), suggesting that ROCK2 is also the major ROCK family member responsible for the phosphorylation of IRF4 in ABC-DLBCL.

To examine the functional relevance of dysregulated ROCK2 activation in ABC-DLBCL, we next performed RNA-seq analysis on U2932 ROCK2 knockdown cells and compared the findings to those in scrambled shRNA controls. We identified 1,391 genes that were differentially expressed (false discovery rate (FDR), q < 0.05) in U2932 ROCK2 knockdown cells compared to scrambled shRNA control cells (Table S1). Over-representation pathway analysis of the differentially expressed genes following ROCK2 silencing revealed that ROCK2 induced genes encoded proteins involved in the regulation of gene expression (such as *TAF1C*, *TFAP2A*, and *ELL*), in DNA repair pathways (such as *XRCC3*, *BRCA1, RAD9A, RAD52, BRCA2,* and *MDC1*), and several cell cycle regulators (including *CDT1*, *CDK11A*, *E2F2*, and *E2F1*) (Figs. 4c, S3B-D). In contrast, ROCK2 repressed the expression of genes encoding proteins involved in B-cell receptor (BCR) (such as *LYN*, *BLNK*, and *FYN*) and co-stimulatory (including *PDCD1*, *CD86*, and *HLA-* family members) signaling pathways (Figs. 4d, S3E-F). Since terminal differentiation requires B-cells to transition from a highly activated state to a plasmacytic phenotype, these findings suggest that ROCK2 may support the PB-like features of ABC-DLBCL by promoting the expression of pathways that allow for the maintenance of proliferation and survival while repressing components of the B-cell activation program, such as BCR signaling and co-stimulation. To explore whether ROCK2 activation is also a feature of primary ABC-DLBCL, we compared the ROCK2-dependent transcriptional profile with the transcriptional profile of 116 primary DLBCL cases obtained from dbGAP (TCGA; accession number phs000235.v6.p1). ROCK2-induced targets were significantly enriched in primary ABC-DLBCL as compared to GCB-DLBCL (Fig. 4e). These data support the idea that dysregulated ROCK2 activity contributes to the transcriptional profile of primary ABC-DLBCL.

To identify transcription factors and epigenetic regulators that could potentially mediate the transcriptional effects observed upon ROCK2 silencing, we performed an upstream regulator enrichment analysis using EnrichR on the ROCK2-regulated geneset using the ENCODE ChIP-seq database^35,36^. This analysis identified several transcription factors known to play key roles in the pathogenesis of ABC-DLBCL. The most highly enriched upstream regulator of the ROCK2-controlled geneset was RELA (Fig. 4f), a canonical member of the NF-κB family of transcription factors whose expression is required for the survival of several lymphomas, including ABC-DLBCL, and whose activity was previously shown to be regulated by the ROCKs^1,2,21^. In addition, the EnrichR analysis identified IRF4 as a top regulator of the ROCK2-regulated geneset (Fig. 4f), with 190 IRF4-regulated genes also differentially expressed following ROCK2 silencing (Table S2). To further assess whether ROCK2 regulated the expression of known IRF4 targets in ABC-DLBCL, the ROCK2-regulated geneset in U2932 was compared with a previously published dataset of IRF4 targets in ABC-DLBCL (Fig. 4g)^8^. We found a significant enrichment of IRF4-repressed, but not IRF4-induced, targets in U2932 ROCK2 knockdown cells compared to scrambled shRNA controls (Fig. 4g-h). Among the IRF4-repressed targets, ROCK2 repressed the expression of several genes including *CIITA*, *IL10*, *BHLHE40*, and *ZEB2* (Fig. 4h-i). Thus, in ABC-DLBCL, the ROCK2-IRF4 pathway modulates the expression of a subset of IRF4 target genes, which primarily encompasses genes that are repressed by IRF4.

### ROCK2 regulates the levels of MYC protein in ABC-DLBCL

**Figure 5:**
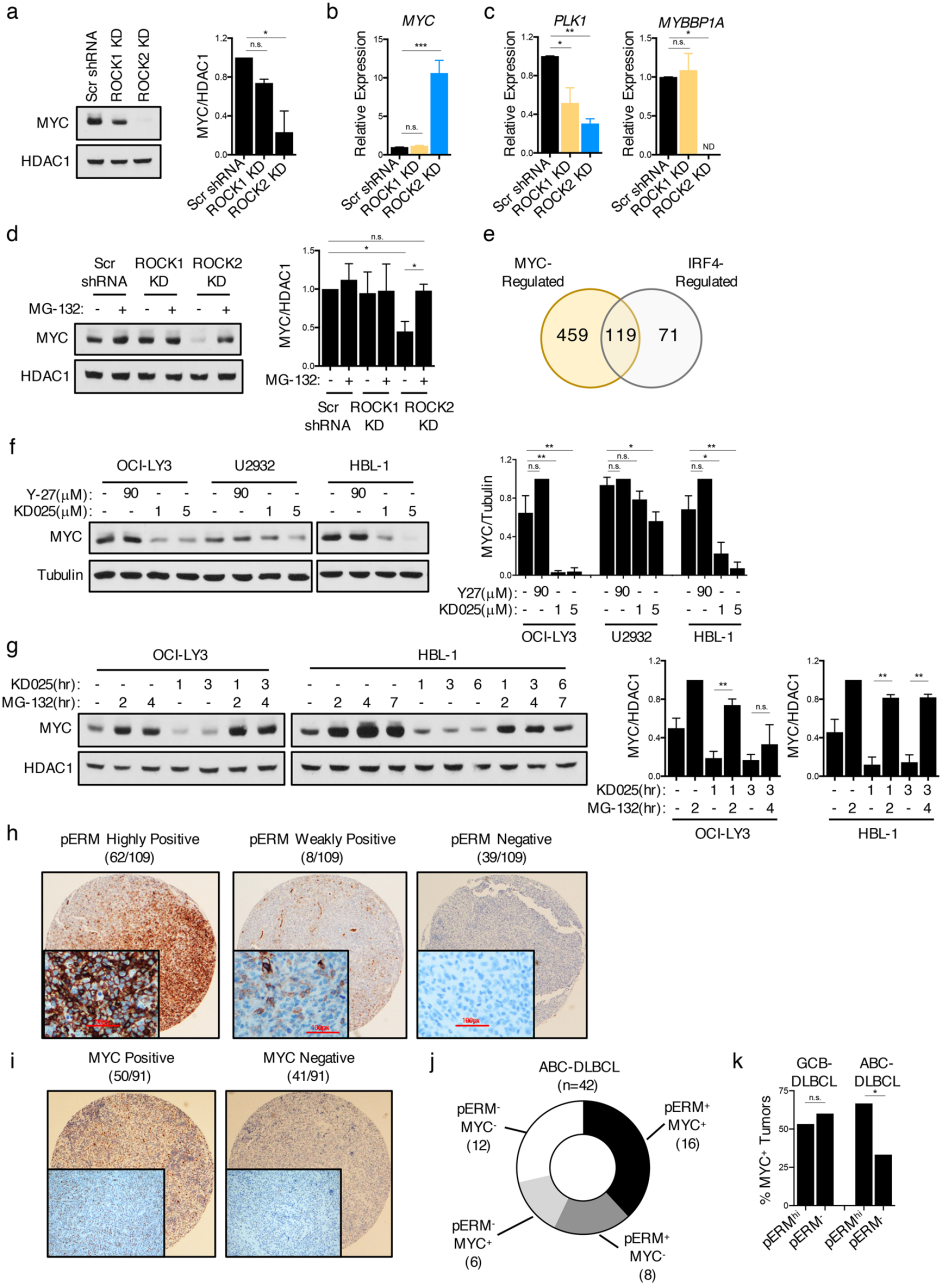
ROCK2 regulates the levels of MYC Protein in ABC-DLBCL. (**a–d**) Stable ROCK knockdowns were generated in U2932 cells following lentiviral infection with shRNA constructs as in Fig. 4. (**a**) Representative immunoblot and quantifications of MYC expression. Quantifications are calculated as the densitometry ratio between MYC to HDAC1 (mean ± SEM; *n* = 2; *p* value by 1-way ANOVA followed by Dunnett’s multiple comparisons test). (**b–c**) Pooled RT-qPCR analysis of indicated genes from U2932 ROCK2 KD *(blue)*, ROCK1 KD *(orange)* and scrambled shRNA control *(black)* cells (mean ± SEM; *n* = 3; *p* value by 1-way ANOVA followed by Dunnett’s multiple comparisons test). (**d**) Representative immunoblot and quantifications of MYC expression from nuclear extracts of U2932 ROCK KD cells left untreated or following treatment with 5 μM MG-132 for 2 h. Quantifications are calculated as the densitometry ratio between MYC to HDAC1 (mean ± SEM; *n* = 3; *p* value by unpaired two-tailed *t* tests). (**e**) Venn diagram showing the overlap of IRF4 and MYC targets from the EnrichR upstream regulator analysis on the U2932 ROCK2-regulated geneset in Fig. 4e. (**f**) Representative immunoblot and quantifications of MYC expression from extracts of DLBCL cells treated for 6 h with Y-27 or KD025 as indicated. Blot separation indicates experiments run on separate gels. Quantifications are calculated as the densitometry ratio between MYC to β-Tubulin (mean ± SEM; *n* = 3; *p* value by 1-way ANOVA followed by Dunnett’s multiple comparisons test). (**g**) Representative immunoblot and quantifications of MYC expression from nuclear extracts of DLBCL cells pre-treated with 5 μM MG-132 for 1 h prior to treatment with 5 μM KD025 for the indicated times. Blot separation indicates experiments run on separate gels. Quantifications are calculated as in (**d**) (mean ± SEM; *n* = 3; *p* value by unpaired two-tailed *t* tests). (**h–i**) Immunohistochemistry of pERM (**h**) and MYC (**I**) on primary DLBCL TMAs. (**J**) Plot showing the frequency of ABC-tumors expressing both pERM and MYC. (**k**) Plot showing the percentage of MYC-positive tumors among pERM^+^ and pERM^-^ tissues in GCB-DLBCL and ABC-DLBCL cases. Statistics based on z-test to compare the total numbers of cases in each subgroup. * p < 0.05, ** p < 0.01, *** p < 0.001, **** p < 0.0001.

In addition to RELA and IRF4, MYC was also identified as a potential upstream regulator of the ROCK2-controlled geneset in U2932 (Fig. 4f; Table S2). To assess the mechanisms by which ROCK2 might control a MYC-regulated transcriptional program, we examined the expression of *MYC* in U2932 ROCK knockdown cells. Given that MYC is well-known to be under complex post-transcriptional regulation^37^, an evaluation of both MYC protein and transcript levels was conducted (Fig. 5a-b). The levels of MYC protein were greatly reduced in U2932 ROCK2 knockdown cells compared to scrambled controls or U2932 ROCK1 knockdown cells (Fig. 5a). In line with the known ability of MYC to negatively regulate its own transcription^38^, the decrease in MYC protein in U2932 ROCK2 knockdown cells was accompanied by higher levels of *MYC* transcripts (Fig. 5b). The decrease in MYC protein levels following silencing of ROCK2 was associated with decreased expression of MYC target genes, including *PLK1* and *MYBBP1A* (Fig. 5c). Pathway analysis of the ROCK2-regulated MYC targets from the EnrichR analysis revealed that these pathways encompassed genes involved in mRNA metabolism, translation, and cell cycle control (Fig. S4A-B; Table S3). Given that degradation via the ubiquitin–proteasome system is one of the crucial mechanisms controlling MYC protein levels^37^, we evaluated whether treatment of U2932 ROCK2 knockdown cells with MG-132, a proteasome inhibitor, could block the downregulation of MYC protein levels observed upon ROCK2 silencing (Fig. 5d). Treatment of U2932 ROCK2 knockdown cells with MG-132 rescued MYC protein expression to levels comparable to those observed in the scrambled shRNA controls or the ROCK1 knockdowns (Fig. 5d). The increase in MYC protein levels did not correspond with increases in *MYC* transcript (Fig. S4C). Interestingly, a comparison between the MYC- and IRF4-regulated genes from the upstream regulator EnrichR analysis revealed substantial overlap (Fig. 5e). These results suggest that ROCK2 can regulate both IRF4 and MYC, albeit by distinct mechanisms, and that IRF4 and MYC functionally cooperate downstream of ROCK2 to promote a common gene signature in ABC-DLBCL.

Given the recent development of selective ROCK2 inhibitors, such as KD025^39^, we next investigated whether the observed effects of ROCK2 on MYC protein stability in ABC-DLBCL could be pharmacologically targeted. To this end, we cultured different DLBCL cell lines with either the selective ROCK2 inhibitor, KD025, or the pan-ROCK inhibitor, Y-27632. Treatment with KD025 resulted in decreased MYC protein levels in the ABC-DLBCL cells examined, while no significant changes in *MYC* transcription were observed at these time points (Figs. 5f, S4D). MYC protein levels were instead not affected by treatment with Y-27632 (Fig. 5f). KD025 treatment also decreased levels of pIRF4 in ABC-DLBCL cells (Fig. S4E). Consistent with our observations in U2932 ROCK2 knockdown cells, proteasome inhibition blocked the decrease in MYC protein observed upon KD025 treatment without an increase in transcript levels (Figs. 5g, S4F). Taken together, these data suggest that ROCK2 supports a MYC-regulated transcriptional program in ABC-DLBCL by promoting the stability of MYC protein and that this pathway can be targeted with selective ROCK2 inhibitors.

Since high levels of ROCK2 activity promote MYC protein expression in ABC-DLBCL cell lines, we next assessed whether hyperactivation of this pathway was also a feature of primary ABC-DLBCL. To this end, we employed immunohistochemistry to examine MYC protein levels and the phosphorylation of ERM (pERM) proteins as a read-out for ROCK activation since staining with the ROCK2-specific pIRF4 antibody was unsuccessful despite multiple attempts. The use of pERM as a marker for ROCK activity in DLBCL cell lines was first validated by intracellular FACS as demonstrated by the finding that the high levels of pERM were significantly decreased following knockdown of ROCK1 or ROCK2 (Fig. S5A). We next stained a panel of primary DLBCL tumors and categorized pERM expression levels as highly positive, weakly positive, or negative (Figs. 5h, S5B). Out of the 109 DLBCL cases examined, 64% exhibited some level of pERM positivity (Figs. 5h, S5B), suggesting that the ROCKs are activated in a subset of primary DLBCL cases. ABC-DLBCL and GCB-DLBCL tumors stained positive for pERM at similar frequencies (Fig. S5C). We next stained this panel of DLBCL tumors for MYC expression. 50/91 DLBCL cases stained positive for MYC expression with a similar distribution of MYC positivity between GCB- and ABC- cases (Figs. 5I, S5D-E). Interestingly, there was a significant association between pERM and MYC expression in the ABC-DLBCL, but not in the GCB-DLBCL cases (Fig. 5j-k). These data thus suggest that a subset of DLBCL patients also exhibit elevated ROCK activity levels.

### ROCK inhibition selectively decreases the survival of ABC-DLBCL

**Figure 6:**
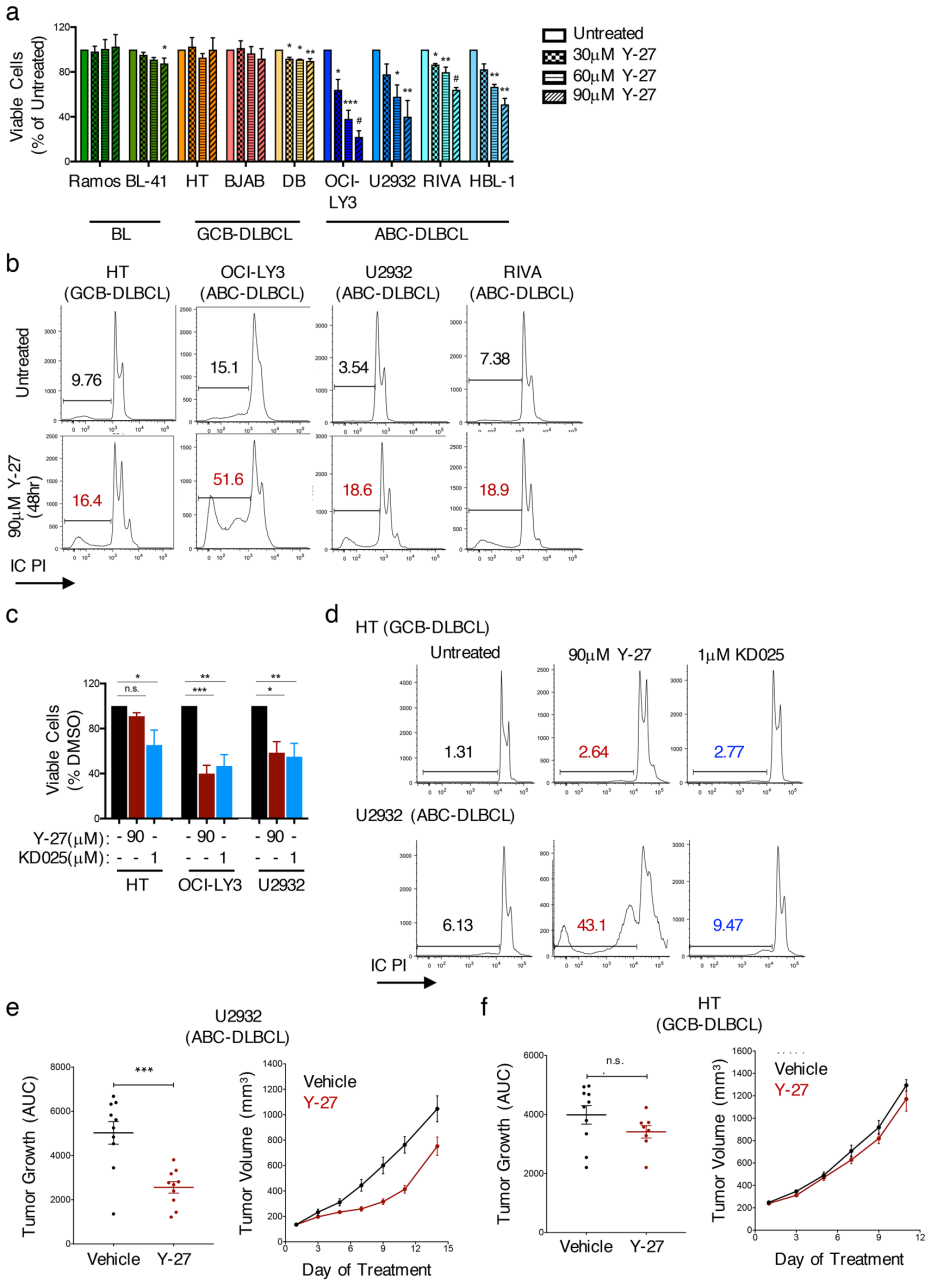
ROCK inhibition selectively decreases the survival of ABC-DLBCL. (**a**) Viability analysis of BL, GCB-DLBCL, and ABC-DLBCL cell lines following 4 days treatment with 0 μM, 30 μM, 60 μM, or 90 μM Y-27 as determined by MTS proliferation assay (mean ± SEM; *n* =  > 2 per cell line; *p* value by 1-way ANOVA followed by Dunnett’s multiple comparisons test). (**b**) Representative histograms of propidium iodide (PI) incorporation showing the sub-G0 cell populations in DLBCL cells either left untreated or following 48 h treatment with 90 μM Y-27. Data representative of 3 independent experiments per cell line. (**c**) MTS proliferation assay of DLBCL cells following 4 days treatment with 90 μM Y-27 or 1 μM KD025 (mean ± SEM; *n* =  > 4 per cell line; *p* value by 1-way ANOVA followed by Dunnett’s multiple comparisons test). (**d**) Representative histograms of PI incorporation in DLBCL cells treated for 48 h with 90 μM Y-27 or 1 μM KD025. Data representative of 3 independent experiments per cell line. (**e–f**) U2932 (**e**) and HT (**f**) cells were established as a subcutaneous tumor in immunodeficient NSG mice and treated daily for 10–15 days with PBS (vehicle) or 40 mg/kg Y-27 by intraperitoneal injection. Tumor progression was monitored as a function of tumor volume. Data pooled from 8–10 mice per treatment condition per cell line. * p < 0.05, ** p < 0.01, *** p < 0.001, ^#^* p* < 0.0001.

Given the ability of ROCK2 to coordinate transcriptional programs known to be involved in the survival of ABC-DLBCL, we next asked whether ROCK inhibition could decrease DLBCL survival. To address this, we cultured ABC-DLBCL, GCB-DLBCL, and BL cells in the presence of increasing doses of Y-27632 and monitored cell viability (Fig. 6a). While the viability of GCB-DLBCL and BL cells was not affected by the pan-ROCK inhibitor, ABC-DLBCL cells exhibited significantly decreased viability following treatment with Y-27632 (Fig. 6a). Treatment of ABC-DLBCL cells with Y-27632 also resulted in an accumulation of non-viable sub-G0 cells and an induction in caspase-3 activity (Figs. 6b, S6A). In contrast to the pan-ROCK inhibitor, treatment with the selective ROCK2 inhibitor, KD025, while decreasing the proliferation of ABC-DLBCL cells to levels comparable to those observed with Y-27632, only minimally affected their survival (Fig. 6c-d). KD025 also exerted a small effect on the viability of a GCB-DLBCL cell line (Fig. 6c). These findings suggest that, while activation of ROCK2 can promote the proliferation of ABC-DLBCL cells, the survival of these cells depends on the activity of both ROCK isoforms.

To further assess the potential of ROCK inhibition as a therapeutic strategy for the treatment of ABC-DLBCL, we employed xenograft models in which an ABC-DLBCL cell line (U2932) or a GCB-DLBCL cell line (HT) was engrafted into immunodeficient NSG mice. Once the tumors started growing, mice were treated with 40 mg/kg Y-27632, a dose demonstrating decreases in IRF4 phosphorylation in vivo (Fig. S6B). Pan-ROCK inhibition resulted in reduced tumor growth in the ABC-DLBCL xenograft but showed no effect in the GCB-DLBCL xenograft (Fig. 6e-f), suggesting that the selective effects of pan-ROCK inhibition on the survival of ABC-DLBCL could also be observed in an in vivo setting. Y-27632 administration did not result in any noticeable toxicities, although Y-27632-treated mice did have a slight increase in serum triglyceride levels compared to vehicle-treated mice, and mouse weight remained normal between the vehicle and Y-27632-treated groups, irrespective of sex (Fig. S6C-D). Thus, ROCK inhibition can selectively suppress the growth of ABC-DLBCL both in vitro as well as in vivo.

### Discussion

ABC-DLBCL is a particularly aggressive B-cell lymphoma subtype characterized by chronic BCR signaling and dysregulations in the molecular networks controlling plasma cell differentiation^1,2^. Here, we have demonstrated that dysregulated activation of ROCK2 is primarily observed in ABC-DLBCL but not in GCB-DLBCL or in BL. ROCK2 furthermore promotes the expression of a unique transcriptional program that supports the phenotype of ABC-DLBCL. At a mechanistic level, ROCK2 regulates the activity of key transcription factors involved in the pathogenesis of ABC-DLBCL through the direct phosphorylation of IRF4 and through the control of MYC protein levels. We further show that inhibition of ROCK signaling decreases survival of ABC-DLBCL. These studies thus uncover a previously unknown role for ROCK2 dysregulation in ABC-DLBCL and suggest that ROCK inhibition could represent a novel therapeutic option for the treatment of these B-cell malignancies.

In contrast to the heightened activation of ROCK2 in ABC-DLBCL, we observed no or only low levels of ROCK2 activity in representative GCB-DLBCL and BL cell lines. Similar to our findings in T-cells^31^, we found that ARHGEF1 activity was associated with ROCK2 activation in B-NHL lines. Interestingly, CD40 engagement alone was sufficient to induce ARHGEF1 activation in Ramos cells, whereas co-stimulation with IL-21 was required for maximal IRF4 phosphorylation. These findings raise the possibility that the combination of these signals may promote changes to the ARHGEF1-containing complex that further enhances ROCK2 activation and IRF4 phosphorylation. Given that inactivating mutations in upstream regulators of the RhoA pathway, including ARHGEF1, have been identified in primary GCB-DLBCL and BL^26–28,40^, our findings present an interesting model whereby fine-tuning the activity of the RhoA-ROCK pathway may be essential to prevent pathophysiology; aberrant activation of ROCK2 may promote ABC-DLBCL, while inactivation of this pathway may contribute to the pathogenesis of GCB-DLBCL or BL.

Our studies identified additional novel mechanisms by which ROCK2 regulates the biology of ABC-DLBCL. Interestingly, one such mechanism involved the regulation of MYC protein levels. MYC expression in DLBCL increases lymphoma aggressiveness and is associated with a poorer clinical prognosis^6,7^. Although MYC is expressed at high levels in many DLBCL, most lack amplifications or translocations of the MYC locus, suggesting that other mechanisms are required to achieve MYC over-expression in ABC-DLBCL. Our findings now suggest that aberrant ROCK2 activation, by blocking the proteasome-dependent degradation of MYC, can promote increased MYC protein levels in ABC-DLBCL potentially leading to its over-expression. Whereas selective ROCK2 inhibition decreased MYC protein levels, pan-ROCK inhibition with Y-27632 showed no effect on MYC protein, suggesting a dynamic interplay between ROCK1 and ROCK2 in promoting MYC expression whereby the ROCK2-mediated effects on MYC require prior and/or concomitant activation of ROCK1. Given that IRF4 was previously identified as a suppressor of MYC-driven leukemias^41^, the ability of ROCK2 to simultaneously promote IRF4 activation and MYC protein expression suggest that the tumor suppressor effects of IRF4 in B-cell leukemias are not dependent on ROCK2-mediated phosphorylation. Bioinformatics analysis also identified other upstream regulators of the ROCK2-regulated geneset in ABC-DLBCLs, including RELA, a member of the NF-κB family of transcription factors, which has previously been shown to be regulated by the ROCKs in several cell types^21^. RELA is activated upon CD40 engagement in B-cells and is required for GC-derived PB/PC formation following immunization^42^. In addition to its physiological roles in PB/PC formation, nuclear RELA has been detected across a variety of B-cell malignancies and NF-κB activation is required for the survival of several lymphomas, including ABC-DLBCL^1,2^. Thus, ROCK2 dysregulation can affect the activity of IRF4, MYC, and RELA, three of the major transcriptional regulators implicated in ABC-DLBCL pathogenesis, indicating that ROCK2-dependent pathways can control the transcriptional landscape of these lymphomas by targeting multiple crucial regulatory hubs.

The selective effects of Y-27632 on the survival of ABC-DLBCL but not GCB-DLBCL suggest that ROCK inhibition could be a potential therapeutic strategy for the treatment of ABC-DLBCL. This notion was further supported by the finding that the transcriptional program affected by ROCK2 silencing in ABC-DLBCL lines was also significantly enriched in primary ABC-DLBCL cases compared to GCB-DLBCL cases. In addition to the marked inhibitory effects of pan-ROCK inhibition on ABC-DLBCLs survival, our studies furthermore raise the possibility that ROCK2 selective inhibitors could be used in combination with other therapeutic agents. Consistent with increases in *CIITA* expression following ROCK2 silencing, our RNA-seq analysis revealed that ROCK2 represses an antigen processing and presentation program in ABC-DLBCL. Loss of MHC-II expression correlates with poor clinical outcome in patients with DLBCL following CHOP or R-CHOP therapies, possibly due to decreased tumor-infiltrating T-cells and thus, a loss in immune surveillance^43^. Selective ROCK2 inhibition could thus be used in combination with current therapeutics to enhance their efficacy.

Our findings outline novel roles for ROCK2 in the regulation of IRF4 and MYC activity in ABC-DLBCLs. Given the high degree of heterogeneity in B-cell malignancies, analysis of clinical specimens will be required to fully appreciate the implications of ROCK2 signaling in ABC-DLBCL. Interestingly, elevated expression of ROCK2 protein has been reported in lymph nodes from Mantle Cell Lymphoma patients^44^, suggesting that that targeting the ROCK2 pathway could also have implications in other B-cell malignancies. In addition to the implications in lymphomagenesis, these findings may carry broader significance across multiple disease settings. Indeed, several of the dysregulated pathways seen in ABC-DLBCLs have also been observed in autoimmune patients including those involving BCR, TLR, and NF-κB signaling, as well as dysregulations in IRF activity^1,10,45^. Additionally, ROCK2 hyperactivation has been observed in several murine models and in human patients with autoimmunity^21^. Dysregulation in ROCK2 activity may thus be a common link contributing to the pathophysiology of several diseases marked by hyperactive B-cell responses.

## Methods

### Cell culture

Cell lines were grown in media to log phase at 37 °C, 5% CO_2_ as follows: Burkitt’s lymphoma cells (BLs; Ramos, BL-41, BL-2), GC B-cell-like (GCB-) diffuse large B-cell lymphoma cells (DLBCL; BJAB, DB, HT), and Activated B-cell like (ABC-) DLBCL cells (OCI-LY3, U2932, RIVA, HBL-1, SU-DHL-2) in Iscove’s DMEM + 10% FBS + Penicillin/Streptomycin. Cell lines were routinely checked for mycoplasma contamination with the MycoAlert Mycoplasma Detection Kit (Lonza). For αCD40 and/or IL-21 stimulations, Ramos cells were plated at 1 × 10^6^ cells/mL and stimulated with 1 μg/mL αCD40 (G28.5; BioLegend) and/or 100 ng/mL IL-21 (Peprotech). For MG-132 experiments, cells were pre-treated with 5 μM MG-132 for 1 h prior to treatment with Y-27632 or KD025. HEK-293 T cells were grown in DMEM + 10% FBS + Penicillin/Streptomycin. 293 T cells were transfected using either standard calcium phosphate procedures or using the TransIT-293 Transfection Reagent (Mirus) according to manufacturer’s protocol.

### Constructs, drugs, and recombinant proteins

Expression plasmids for MYC- and FLAG-tagged human IRF4 were generated as previously described^19^. The compound Y-27632 was obtained from EMD Millipore and KD025 was obtained from SelleckChem. NP-conjugated chicken gamma-globulin (CGG) was obtained from Biosearch Technologies. MG-132 was obtained from Sigma.

### MTS proliferation assays

Cells were plated in triplicate at a density of 5,000–10,000 cells per well in 96-well plates and treated for 4 days with increasing doses of KD025 or Y-27632. For Y-27632 treatments, cells were boosted with a second dose of inhibitor at day 2. Cell viability was assayed by adding 3-(4,5-dimethylthiazol-2-yl)-5-(3-carbpoxymethoxyphenol)-2-(4-sulphophenyl)-2H tetrazolium and an electron-coupling reagent (Promega), incubated for 3 h and measured by the amount of 490 nm absorbance using a 96-well plate reader. The background was subtracted using media only controls.

### Flow cytometry

For intracellular staining, cells were fixed and permeabilized with the Foxp3 Staining Buffer Set (eBioscience) according to the manufacturer’s instructions. Cells were stained with antibodies against IRF4 (200×; 3E4; eBioscience) or activated caspase-3 (C92-605; BD) for 30 min on ice. For assessment of the dead sub-G0 population, cells were incubated with 50 μg/mL propidium iodide (PI) in the presence of 100 μg/mL RNaseA. For pERM staining, cells were fixed and permeabilized with Cytofix/Cytoperm buffer (BD) and washed twice with Perm/Wash buffer (BD). The cells were stained with an antibody against pERM (250×; 48G2; Cell Signaling) for 40 min at room temperature. Primary antibody was detected with anti-rabbit antibodies (Invitrogen). All flow cytometry data was acquired on a FACS Canto (BD) and analyzed with FlowJo (TreeStar) software.

### Lentiviral infection and generation of stable cell lines

Lentiviral infections were performed using a 3rd generation packaging system. MISSION shRNA expression constructs targeting ROCK1 and ROCK2 were generated in pLKO.1 vectors containing puromycin resistance cassettes and either tagRFP (for ROCK1 shRNA) or tGFP (for ROCK2 and scrambled shRNAs) cassettes and were obtained from Sigma. Lentiviral particles were produced by transient transfection of 293 T as previously described^8^. The sequences for the shRNAs are shown in Supplementary Table S4.

### RNA extraction and RT-qPCR

Total RNA was isolated from cells using the RNeasy Plus Mini kit (Qiagen). cDNAs were prepared using the iScript cDNA synthesis kit (BioRad). Real-time quantitative PCR was performed using the iTaq Universal SYBR Green Supermix (BioRad). Primer sequences are found in Supplementary Table S4.

### RNA-seq analysis

Nextera libraries were prepared and paired-end sequenced by the Weill Cornell Epigenomics Core using HiSeq2500 at a depth of ~ 30–50 million fragments per sample. Quality of library preparations was evaluated by BioAnalyzer 2,100 (Agilent). Raw sequencing data was evaluated using FASTQC and 50-bp paired reads were mapped to a human reference genome (version hg19 from UCSC) using STAR (version 2.4.2) aligner^46,47^. Aligned reads were then quantified using *HTSeq* for raw counts and *Cufflinks* (version 2.2.1) for FPKM. To classify the samples based on gene expression profiles, hierarchical data clustering and principal component analysis were performed on the log_2_ transformed FPKM expression values in R statistical software. Prior to differential expression analysis, R package *ComBat* was used to correct for replicate-based batch effect. Differential expression analysis was performed on normalized batch-corrected raw counts using the *limma* package in R. Genes with false discover rate (FDR), q < 0.05 were considered to be significantly differentially expressed.

Gene set enrichment analysis was performed using GSEA software (Broad Institute)^48^. Genes were ranked by the t-statistic value obtained from comparisons and the pre-ranked version of the tool was used to identify significantly enriched biological pathways. Gene sets enriched with FDR q < 0.20 were considered significant. Additionally, gene-name based pathway analysis was carried out using the online webtool ConsensusPathDB (CPDB).

### Immunoblotting, kinase activity assays, and GEF pulldowns

Cell extracts were prepared as previously described^19^. The purity of nuclear and cytoplasmic fractions was verified by probing with antibodies against LaminB1 (D4Q4Z; Cell Signaling) or HDAC1 (#2062; Cell Signaling), and β-tubulin (D66; Sigma). Antibodies against ARHGEF1 (H-165), BLIMP1 (6D3), MYC (9E10), IRF4 (M-17), IRF8 (C-19), ROCK1 (H-85), and ROCK2 (H-85) were obtained from Santa Cruz Biotechnology. Antibodies against pSTAT3 (Y705, #9,131) were obtained from Cell Signaling. Antibodies against STAT3 (84) were obtained from BD Biosciences. Rabbit polyclonal antibody specific for pIRF4 (recognizing phosphorylated S446/S447 residues) was generated as previously described^19^. ARHGEF1 pulldowns were performed as previously described^31^. ROCK kinase activity assays were performed following immunoprecipitation of ROCK1 (C-19; Santa Cruz) or ROCK2 (C-20; Santa Cruz) from nuclear extracts as previously described^19,31^. For protein–protein interaction studies, cell extracts were immunoprecipitated with an anti-FLAG agarose affinity matrix (Sigma) according to manufacturer’s instructions. Immunoblot images were prepared using Adobe Photoshop and quantifications were calculated using ImageJ software.

### Chromatin immunoprecipitation (ChIP) and oligonucleotide precipitation (ONP) assays

For chromatin immunoprecipitation (ChIP) assays, cells were harvested and chromatin extracts were prepared using the truChIP Chromatin Shearing Reagent Kit (Covaris) according to the manufacturer’s instructions. 100 μg of the sonicated DNA–protein complexes was used for immunoprecipitions with anti-IRF4 (M-17; Santa Cruz) or normal goat Ig control (Santa Cruz) antibodies. After cross-linking was reversed and proteins were digested, the DNA was purified from the immunoprecipitates as well as from input extracts and then was analyzed by qPCR. Primer sequences are described in Supplementary Table S4.

Oligonucleotide precipitation assays (ONPs) were conducted as previously described^19^. In brief, nuclear extracts were incubated with biotinylated double-stranded oligonucleotides corresponding to amplified IRF4-bound ChIP regions. Proteins bound to the biotin-labeled DNA were collected by streptavidin-agarose beads, separated by 10% SDS-PAGE and analyzed by immunoblot with anti-IRF4 (D9P5H; Cell Signaling) antibodies. Sequences of oligonucleotides are described in Supplementary Table S4.

### Mice, immunizations, and cell sorting

Blimp1-YFP reporter mice were obtained from E. Meffre (Yale University, New Haven, CT). For immunization experiments, 8–10 week old mice were immunized with 100 μg of NP_30-40_-CGG (Biosearch Technologies) precipitated in alum. For cell sorting, single-cell suspensions were pooled from spleens of immunized mice and were enriched for B-cells and plasmablasts using biotinylated B220 (RA3-6B2; BD) and CD138 (281–2; BD) antibodies and streptavidin microbeads (Miltenyi Biotech). Plasmablasts (Blimp1-YFP^+^CD138^+^) and follicular B-cells (B220^+^Blimp1-YFP^-^CD23^+^) were sorted on a FACSAriaII. All the mice used in the experiments were housed in a specific pathogen-free animal facility at Weill Cornell Medical College. Experiments were performed according to the protocols approved by the Institutional Animal Care and Use Committee of Weill Cornell Medicine.

The xenograft tumor models of human ABC-DLBCL and GCB-DLBCL were established by subcutaneous (s.c.) injection of 10–20 × 10^6^ U2932 or HT cells into the flank of male and female NOD/SCID gamma (NSG) mice. The tumor growth was monitored every other day by measuring size in two orthogonal dimensions with an electronic digital caliper. Tumor volume (mm^3^) was calculated by using the formula ½ (long dimension)  × (short dimension)^2^. When tumors reached a palpable size (approximately 75–100 mm^3^), mice were randomized into two treatment groups. The mice were treated intraperitoneally everyday with vehicle alone (PBS) or Y-27632 (40 mg/kg). Mice were weighed twice per week and all mice were euthanized when at least two tumors reached the maximal tumor mass permitted by our protocol, which was between days 12–15 after initiation of therapy. At the moment of euthanasia, blood was collected and tumors were harvested. Experiments were performed according to the protocols approved by the Institutional Animal Care and Use Committee of Weill Cornell Medicine.

### Tissue microarray (TMA) immunohistochemistry

Immunohistochemistry (IHC) was applied to a tissue microarray (TMA) encompassing 109 DLBCL tissues using a rabbit monoclonal antibody against pERM (16,000×; 48G2; Cell Signaling) and MYC (Y69; Abcam). pERM expression was evaluated based on the degree of positivity (highly positive, weakly positive, or negative) and MYC expression was evaluated based on a cutoff of 20% positive cells. Classification of DLBCL tumors as GCB (CD10^+^BCL6^+^MUM1^+/−^) or non-GCB (ABC-; CD10^-^BCL6^+/−^MUM1^+^) was completed using the Hans classification system.

### Statistics

Calculations of *p* values are described in the figure legends and were performed using unpaired two-tailed *t* tests for two-group comparisons or by using one-way ANOVA followed by Tukey’s test for multiple comparisons. Significance was determined by *p* < 0.05 and the following values were delineated: *p < 0.05, **p < 0.01, ***p < 0.001, ****p < 0.0001. Statistical analyses were performed using Graphpad Prism 7.

## Supplementary information

Supplementary Figures.

Supplementary Table S1.

Supplementary Table S2.

Supplementary Table S3.

Supplementary Table S4.

Supplementary Table Legends.

## Data Availability

The data that support the findings of this study are available from the corresponding author upon request. The RNA-seq data have been deposited at accession number GSE147521.
