# Supplementary material for: Selective dysregulation of ROCK2 activity promotes aberrant transcriptional networks in ABC diffuse large B-cell lymphoma
Source: Sci Rep. 2020 Aug 4;10:13094. doi: 10.1038/s41598-020-69884-1 (PMC7403583; doi:10.1038/s41598-020-69884-1)
Supplement: Supplementary file 1 — Supplementary Figures. [file 41598_2020_69884_MOESM1_ESM.pdf]

Selective dysregulation of ROCK2 activity promotes aberrant transcriptional networks in ABC diffuse large B cell lymphoma

Edd Ricker[1,2], Akanksha Verma[3], Rossella Marullo[4], Sanjay Gupta[1], Chao Ye[1], Tania Pannellini[5], Michela Manni[1], Wayne Tam[6], Giorgio Inghirami[6], Olivier Elemento[3], Leandro Cerchietti[4], and Alessandra B. Pernis*[1, 2, 7, 8]

[1]Autoimmunity and Inflammation Program, Hospital for Special Surgery, NY, NY, USA

[2]Graduate Program in Immunology and Microbial Pathogenesis, Weill Cornell Graduate School of Medical Sciences, NY, NY, USA

[3]Caryl and Israel Englander Institute for Precision Medicine, Department of Physiology and Biophysics, Weill Cornell Medicine, NY, NY, USA

[4]Hematology and Oncology Division, Weill Cornell Medicine, NY, NY, USA

[5]Research Division and Precision Medicine Laboratory, Hospital for Special Surgery, NY, NY, USA

[6]Department of Pathology and Laboratory Medicine, Weill Cornell Medicine, NY, NY, USA

[7]David Z. Rosensweig Genomics Research Center, Hospital for Special Surgery, NY, NY, USA

[8]Department of Medicine, Weill Cornell Medicine, NY, NY, USA

Correspondence:

*Alessandra B. Pernis
Autoimmunity and Inflammation Program, Hospital for Special Surgery, 535 East 70th Street, New York, NY 10021, USA. Tel: 212-206-1612. E-mail: pernisa@hss.edu

Figure S1.

a

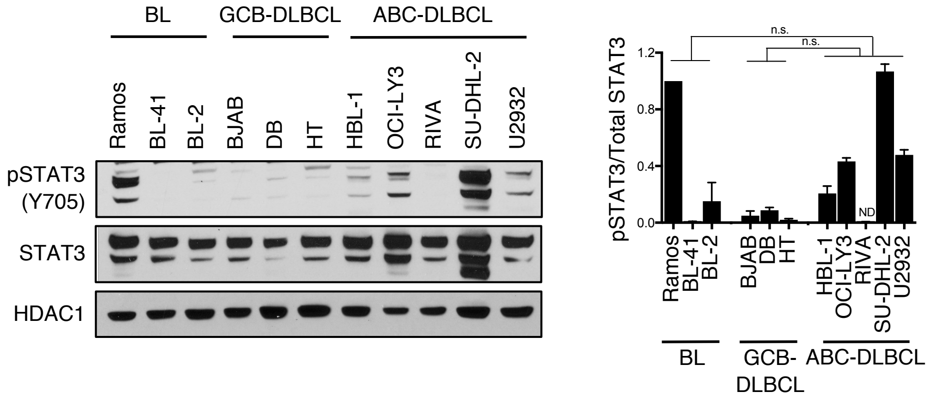

Figure S1. (A) Representative immunoblot and quantifications of phosphorylated STAT3 (pSTAT3; Y705), total STAT3, and HDAC1 as a loading control from nuclear extracts of BL (Ramos, BL-41, BL-2), GCB-DLBCL (BJAB, DB, HT), and ABC-DLBCL (HBL-1, OCI-LY3, RIVA, SU-DHL-2, U2932) cell lines. Quantifications are calculated as the densitometry ratio between pSTAT3 to total STAT3 (mean +/- SEM, n=2; p-value by 1-way ANOVA followed by Tukey's multiple comparisons test). ns $p>0.05$.

Figure S2.

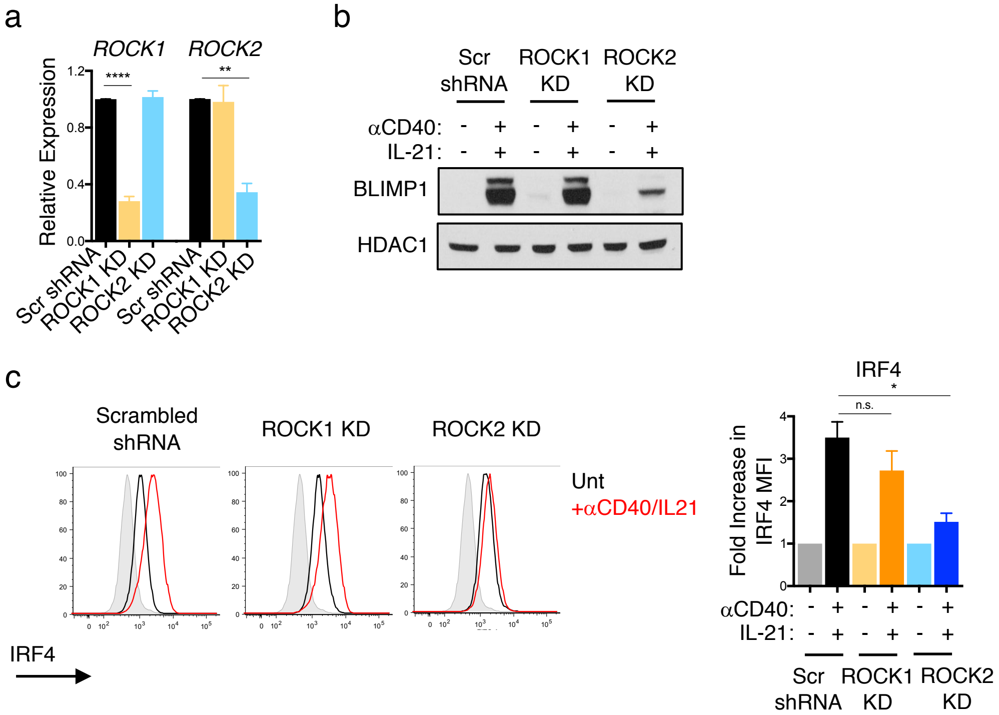

Figure S2. (A) Pooled RT-qPCR analysis of *ROCK1* and *ROCK2* transcripts relative to *28S* in Ramos cells after stable lentiviral infection with shRNA constructs as in Fig. 3 (mean +/- SEM; *n*=3; p-value by 1-way ANOVA followed by Dunnett's multiple comparisons test). **(B-C)** Stable Ramos ROCK1 KD, ROCK2 KD, and scrambled shRNA control cells were left untreated or stimulated for 6hr with αCD40 and IL-21. **(B)** Representative immunoblot of BLIMP1 and HDAC1 as a loading control. Immunoblot representative of 2 independent experiments. **(C)** Representative histograms and quantifications of IRF4 expression (mean +/- SEM; *n*=2; p-value by 1-way ANOVA followed by Dunnett's multiple comparisons test). *p<0.05, **p<0.01, ***p<0.001, ****p<0.0001.

Figure S3.

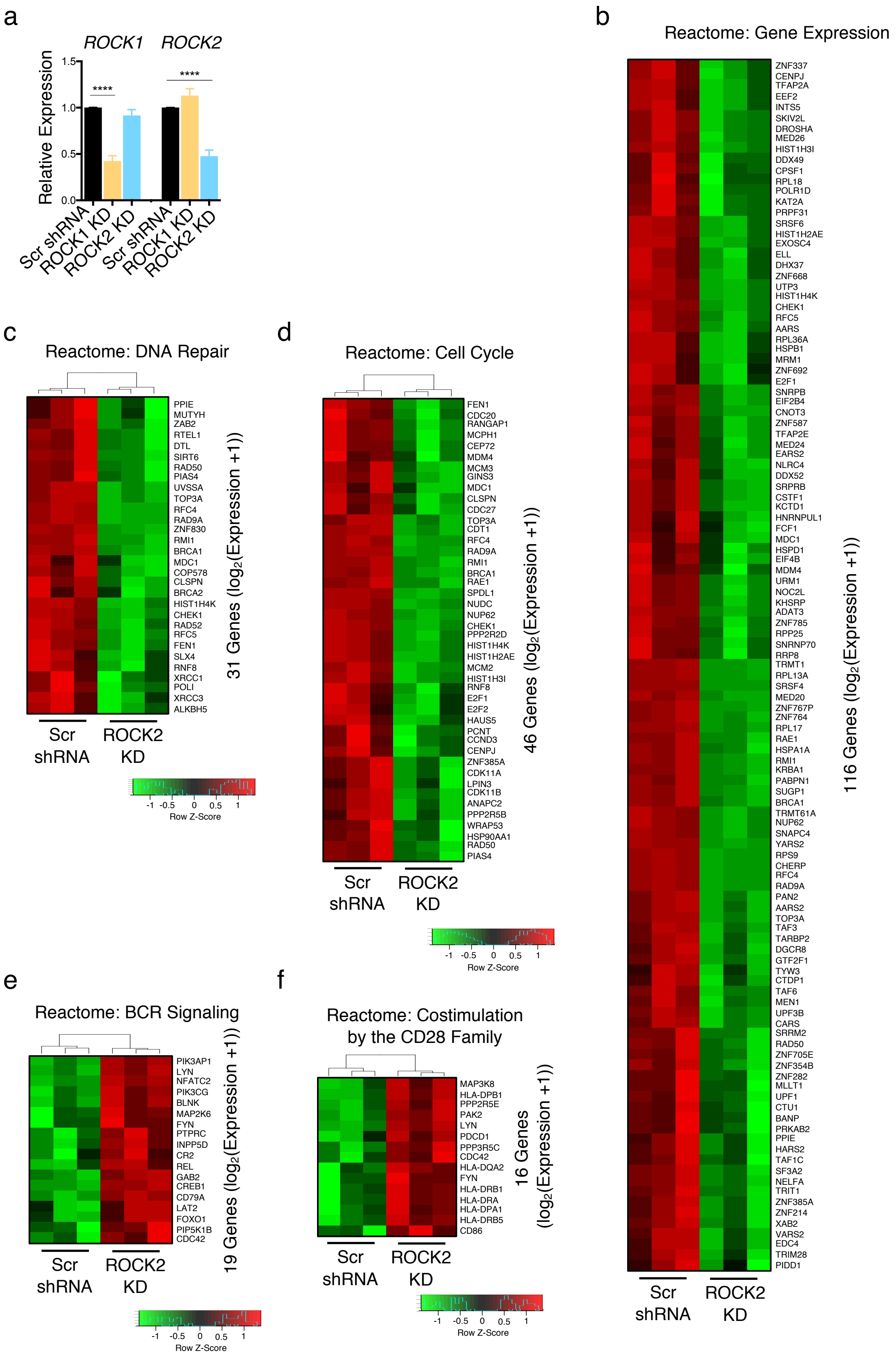
a
ROCK1
ROCK2
Relative Expression
1.5
1.0
0.5
0.0
****
****
Scr shRNA
ROCK1 KD
ROCK2 KD
Scr shRNA
ROCK1 KD
ROCK2 KD
b
Reactome: Gene Expression
ZNF337
CENPJ
TFAP2A
EEF2
INTS5
SKIV2L
DROSHA
MED26
HIST1H3I
DDX49
CPSF1
RPL18
POLR1D
KAT2A
PRPF31
SRSF6
HIST1H2AE
EXOSC4
ELL
DHX37
ZNF668
UTP3
HIST1H4K
CHEK1
RFC5
AARS
RPL36A
HSPB1
MRM1
ZNF692
E2F1
SNRPB
EIF2B4
CNOT3
ZNF587
TFAP2E
MED24
EARS2
NLRC4
DDX52
SRPRB
CSTF1
KCTD1
HNRNPUL1
FCF1
MDC1
HSPD1
EIF4B
MDM4
URM1
NOC2L
KHSRP
ADAT3
ZNF785
RPP25
SNRNP70
RRP8
TRMT1
RPL13A
SRSF4
MED20
ZNF767P
ZNF764
RPL17
RAE1
HSPA1A
RMI1
KRBA1
PABPN1
SUGP1
BRCA1
TRMT61A
NUP62
SNAPC4
YARS2
RPS9
CHERP
RFC4
RAD9A
PAN2
AARS2
TOP3A
TAF3
TARBP2
DGCR8
GTF2F1
TYW3
CTDP1
TAF6
MEN1
UPF3B
CARS
SRRM2
RAD50
ZNF705E
ZNF354B
ZNF282
MLLT1
UPF1
CTU1
BANP
PRKAB2
PPIE
HARS2
TAF1C
SF3A2
NELFA
TRIT1
ZNF385A
ZNF214
XAB2
VARS2
EDC4
TRIM28
PIDD1
116 Genes (log2(Expression +1))
Scr shRNA
ROCK2 KD
c
Reactome: DNA Repair
PPIE
MUTYH
ZAB2
RTEL1
DTL
SIRT6
RAD50
PIAS4
UVSSA
TOP3A
RFC4
RAD9A
ZNF830
RMI1
BRCA1
MDC1
COP578
CLSPN
BRCA2
HIST1H4K
CHEK1
RAD52
RFC5
FEN1
SLX4
RNF8
XRCC1
POLI
XRCC3
ALKBH5
31 Genes (log2(Expression +1))
Scr shRNA
ROCK2 KD
-1 -0.5 0 0.5 1
Row Z-Score
d
Reactome: Cell Cycle
FEN1
CDC20
RANGAP1
MCPH1
CEP72
MDM4
MCM3
GINS3
MDC1
CLSPN
CDC27
TOP3A
CDT1
RFC4
RAD9A
RMI1
BRCA1
RAE1
SPDL1
NUDC
NUP62
CHEK1
PPP2R2D
HIST1H4K
HIST1H2AE
MCM2
HIST1H3I
RNF8
E2F1
E2F2
HAUS5
PCNT
CCND3
CENPJ
ZNF385A
CDK11A
LPIN3
CDK11B
ANAPC2
PPP2R5B
WRAP53
HSP90AA1
RAD50
PIAS4
46 Genes (log2(Expression +1))
Scr shRNA
ROCK2 KD
-1 -0.5 0 0.5 1
Row Z-Score
e
Reactome: BCR Signaling
PIK3AP1
LYN
NFATC2
PIK3CG
BLNK
MAP2K6
FYN
PTPRC
INPP5D
CR2
REL
GAB2
CREB1
CD79A
LAT2
FOXO1
PIP5K1B
CDC42
19 Genes (log2(Expression +1))
Scr shRNA
ROCK2 KD
-1 -0.5 0 0.5 1
Row Z-Score
f
Reactome: Costimulation by the CD28 Family
MAP3K8
HLA-DPB1
PPP2R5E
PAK2
LYN
PDCD1
PPP3R5C
CDC42
HLA-DQA2
FYN
HLA-DRB1
HLA-DRA
HLA-DPA1
HLA-DRB5
CD86
16 Genes (log2(Expression +1))
Scr shRNA
ROCK2 KD
-1 -0.5 0 0.5 1
Row Z-Score

Figure S3. (A) Pooled RT-qPCR analysis of *ROCK1* and *ROCK2* expression relative to *28S* in U2932 cells following lentiviral infections as in Fig. 4 (mean +/- SEM; *n*=5; p-value by 1-way ANOVA followed by Dunnett's multiple comparisons test). **(B-F)** Heat map depictions of differentially expressed genes in the U2932 ROCK2 KD cells compared to the scrambled shRNA control cells composing the gene expression (B), DNA repair (C), cell cycle (D), BCR signaling (E), and costimulation by the CD28 family (F) pathways from the over-representation pathway analysis in Fig. 4C-D. *p<0.05, **p<0.01, ***p<0.001, ****p<0.0001.

Figure S4.

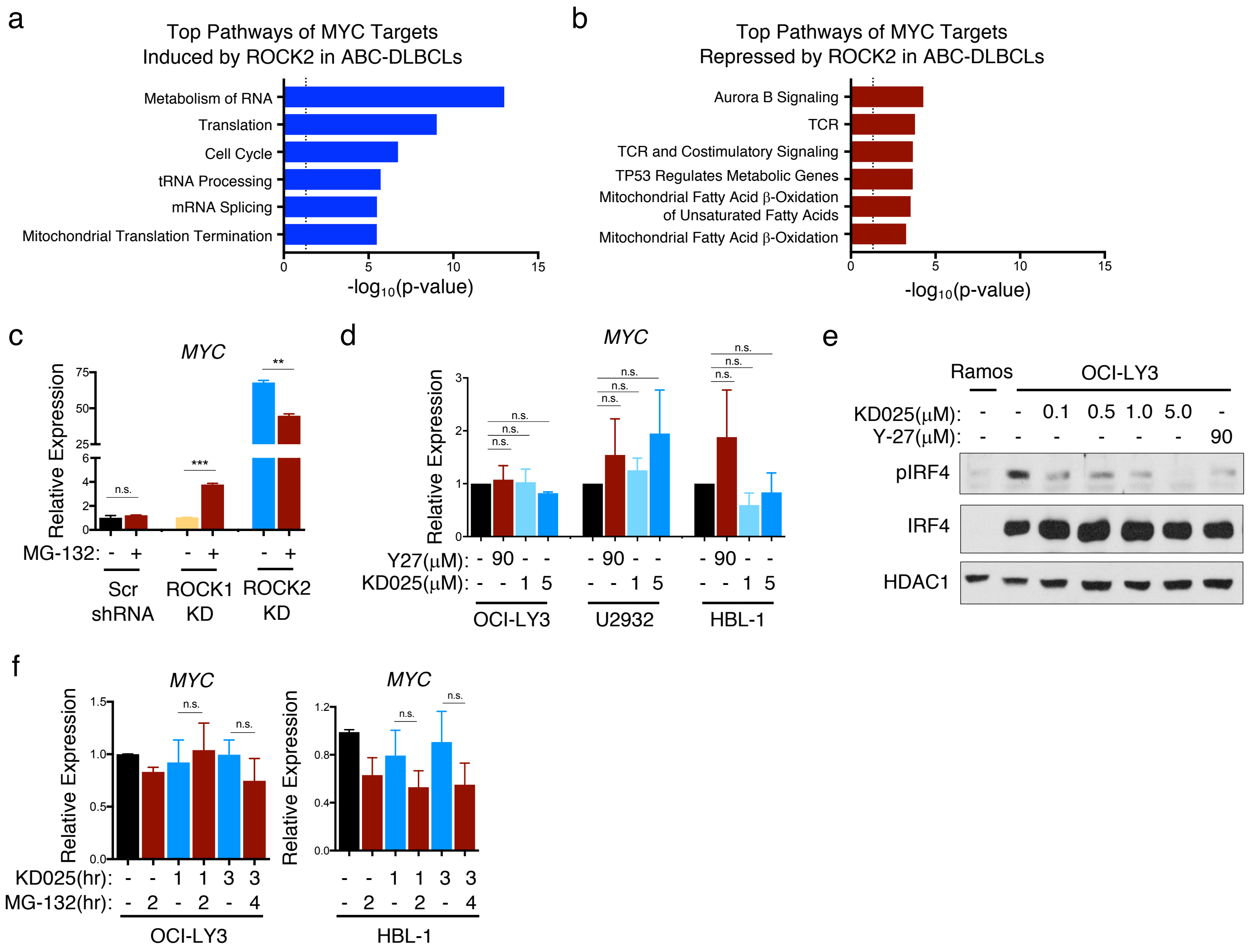

Figure S4. (A-B) Plots showing the -$\log_{10}$(p-value) values for the top over-represented pathways from the genes representing the ROCK2-induced (A) or the ROCK2-repressed (B) MYC targets in U2932. Dotted lines indicate significance cutoffs at p=0.05. **(C)** Representative RT-qPCR analysis for *MYC* transcripts compared to *28S* from the experiment in Fig. 5C. Data representative of 2 independent experiments (mean +- SD; p-value by unpaired two-tailed t-test). **(D)** Pooled RT-qPCR analysis of *MYC* (mean +/- SEM; *n*=3; p-value by 1-way ANOVA followed by Dunnett's multiple comparisons test). **(E)** Representative immunoblot of pIRF4, total IRF4, and HDAC1 in OCI-LY3 cells treated for 6hr with Y-27 or KD025. Data representative of 2 independent experiments. **(F)** Pooled RT-qPCR analysis of *MYC* transcripts relative to *28S* from the experiment in Fig. 5G (mean +/- SEM; *n*=3; p-value by unpaired two-tailed t-tests). *p<0.05, **p<0.01, ***p<0.001, ****p<0.0001.

Figure S5.

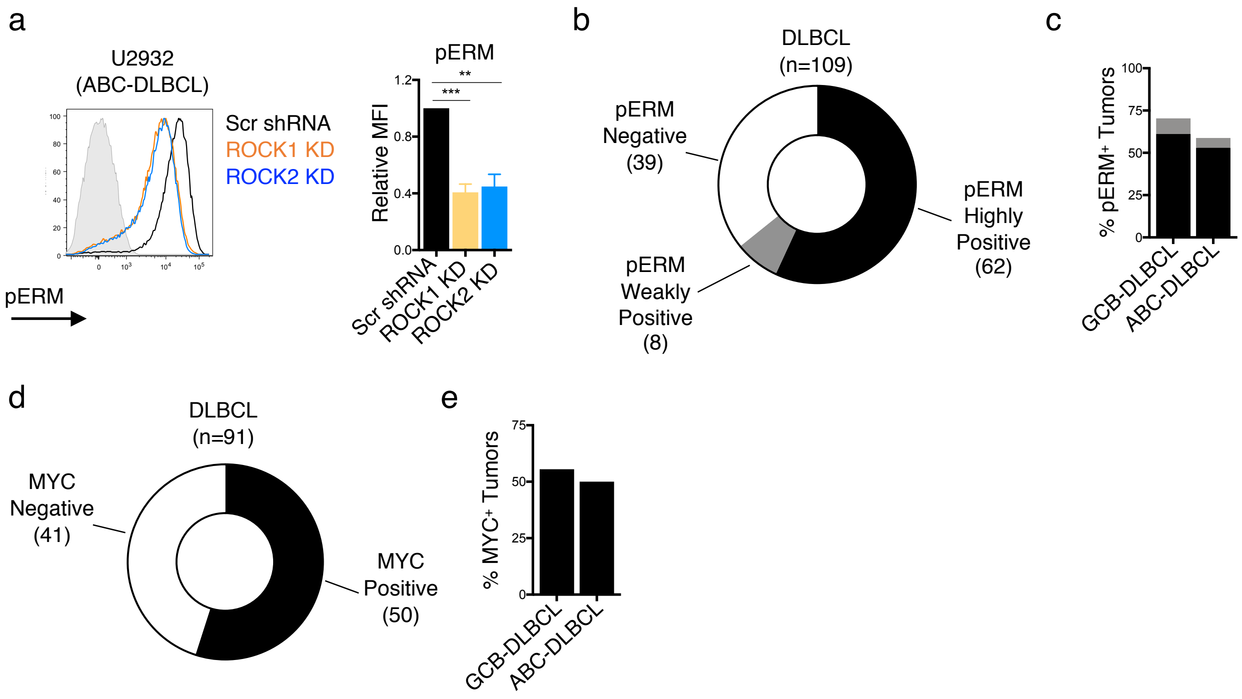

Figure S5. (A) Representative histogram and quantifications of pERM expression in U2932 cells following knockdown of ROCK1 *(orange)*, ROCK2 *(blue)*, or following infection with a scrambled shRNA control construct *(black)* (mean +/- SEM; *n*=3; p-value by 1-way ANOVA followed by Dunnett's multiple comparisons test). **(B)** Plot showing the fraction of highly positive, weakly positive, and negative pERM levels in the total DLBCL cases from Fig. 5H. **(C)** Plot showing the frequency of pERM highly positive *(black)* and weakly positive *(grey)* tumors among GCB- and ABC-DLBCL cases from Fig. 5H. **(D)** Plot showing the frequency of MYC-positive tumors of total DLBCL cases from Fig. 5I. **(E)** Plot showing the frequency of MYC-positive tumors among GCB- and ABC-DLBCL cases from Fig. 5I. $^*p<0.05$, $^{**}p<0.01$, $^{***}p<0.001$, $^{****}p<0.0001$.

Figure S6.

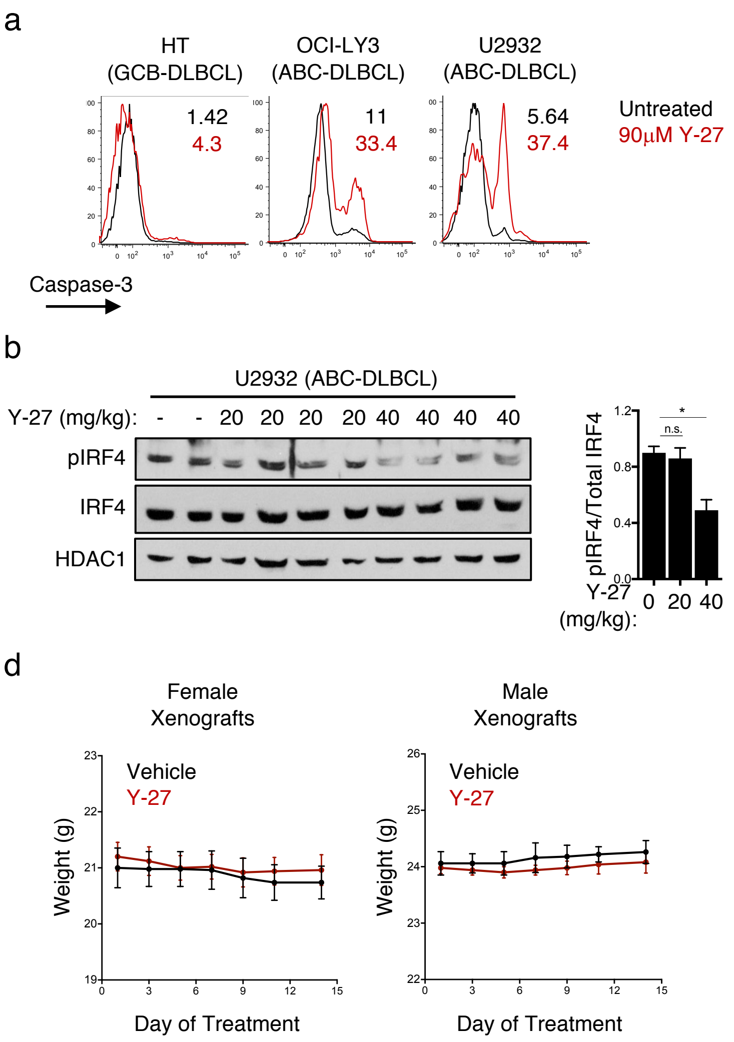

c

Murine Chemical Panel

Parameter	Vehicle Treatment	Y-27632 Treatment
BUN (mg/dL)	17.4 +/- 2.2	19.2 +/- 2.2
CREA (mg/dL)	0.21 +/- 0.048	0.21 +/- 0.065
BUN/CREA Ratio	84.6 +/- 17	96.2 +/- 24
ALP (U/L)	63.8 +/- 37	53.2 +/- 37
ALT (U/L)	64.4 +/- 60	127 +/- 112
AST (U/L)	354 +/- 370	575 +/- 760
GGT (U/L)	0 +/- 0	0 +/- 0
TBIL (mg/dL)	0.58 +/- 0.59	0.52 +/- 0.47
DBIL (mg/dL)	0.08 +/- 0.08	0.04 +/- 0.055
IBIL (mg/dL)	0.5 +/- 0.62	0.48 +/- 0.48
TP (g/dL)	5.72 +/- 0.51	5.9 +/- 0.37
ALB (g/dL)	3.36 +/- 0.20	3.38 +/- 0.16
GLOB (g/dL)	2.36 +/- 0.31	2.52 +/- 0.31
A/G Ratio	1.433 +/- 0.097	1.36 +/- 0.16
P (mg/dL)	13.9 +/- 0.98	13.7 +/- 2.4
Ca (mg/dL)	10.9 +/- 0.69	10.6 +/- 0.48
GLU (mg/dL)	283 +/- 130	251 +/- 100
CHOL (mg/dL)	116 +/- 43	131 +/- 37
TRIG (mg/dL)*	105 +/- 18	149 +/- 23
CK (U/L)	554 +/- 330	931 +/- 900
TCO2 (mEq/L)	19 +/- 5.4	16.4 +/- 7.0
Na (mEq/L)	152 +/- 3.7	153 +/- 6.3
K (mEq/L)	14.9 +/- 5.6	14.3 +/- 6.2
Cl (mEq/L)	104 +/- 6.1	105 +/- 7.3
Na/K Ratio	11.6 +/- 4.9	12.5 +/- 5.2
Anion Gap	44.1 +/- 13.3	45.3 +/- 14

Figure S6. (A) Representative histograms of active cleaved caspase-3 in DLBCL cells either left untreated *(black)* or following 24hr treatment with 90μM Y-27 *(red)*. Data representative of 3 independent experiments per cell line. **(B-C)** U2932 and HT cells were established as subcutaneous tumors in immunodeficient NSG mice as in Fig. 6E-F. **(B)** Immunoblot analysis and quantifications of phosphorylated IRF4 at S446/S447 (pIRF4), total IRF4, and HDAC1 as a loading control from nuclear extracts of U2932 xenografts treated with 20-40mg/kg Y-27 for 24hr by intraperitoneal injection (mean +/- SEM; p-value by 1-way ANOVA followed by Dunnett's multiple comparisons test). **(C)** Table showing the results of a biochemistry panel on the serum from 5 vehicle-treated and 5 Y-27-treated xenografts collected at the termination of the experiment from Fig. 6E-F (mean +/- SD). **(D)** Weight of tumor-implanted female and male mice following treatment with either PBS (vehicle) or Y-27 as in Fig. 6E-F. Data pooled from 8-10 mice per treatment condition per cell line. *p<0.05, **p<0.01, ***p<0.001, ****p<0.0001.

Full Uncut Gels:

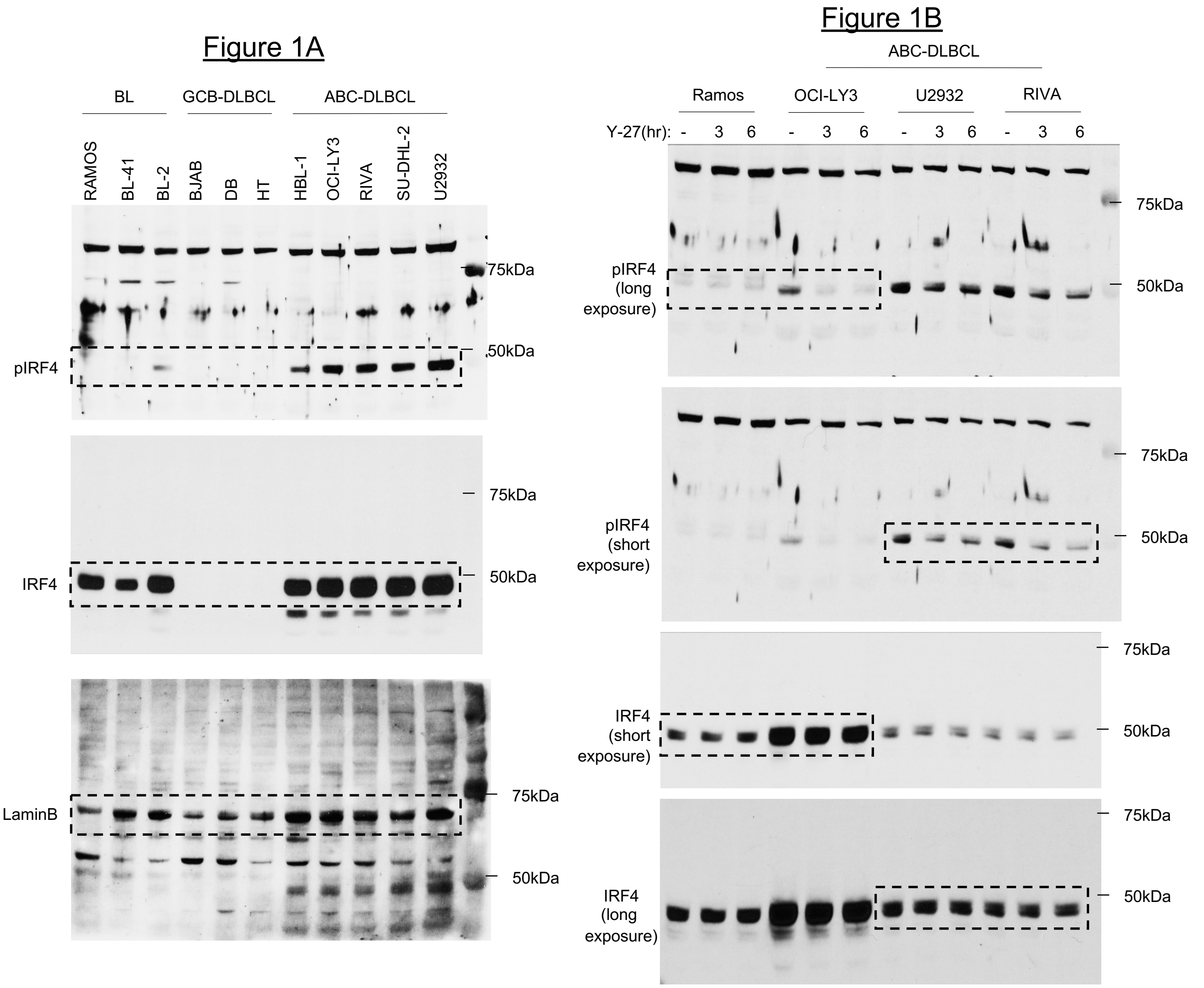

Full Uncut Gels:

Figure 1D

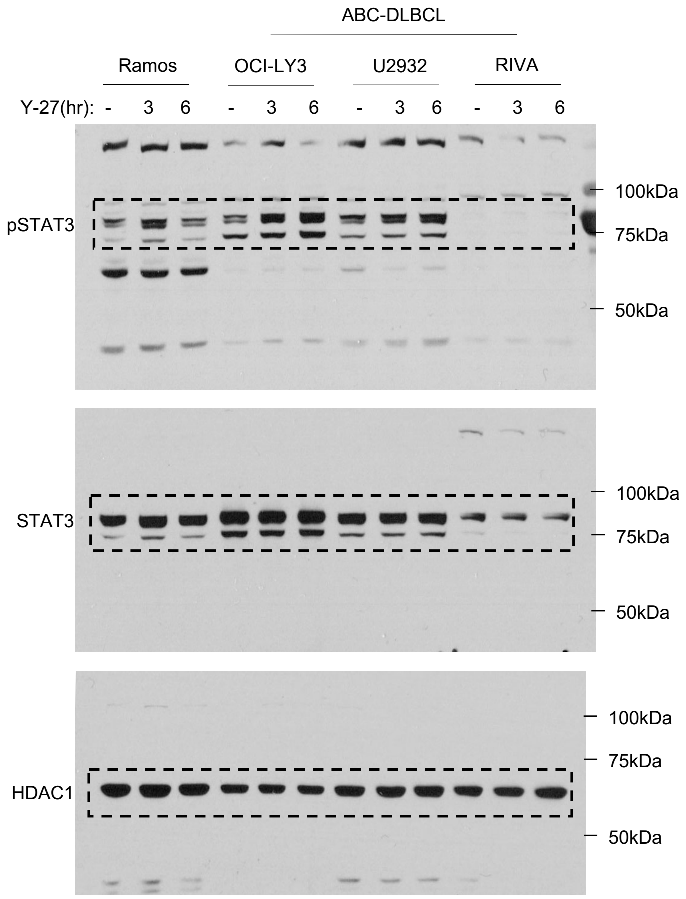

Figure 1E

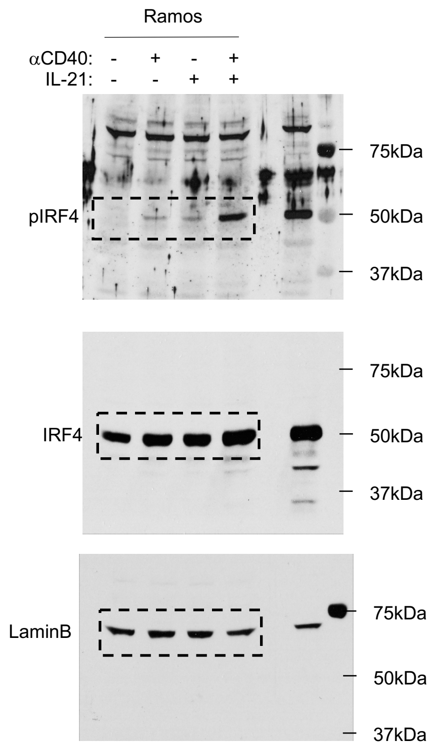

Figure 1F

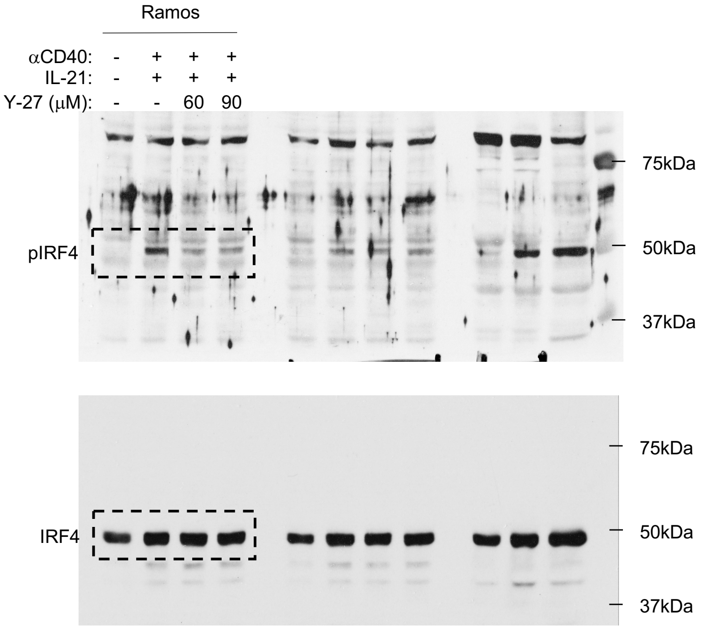

Full Uncut Gels:

Figure 1G

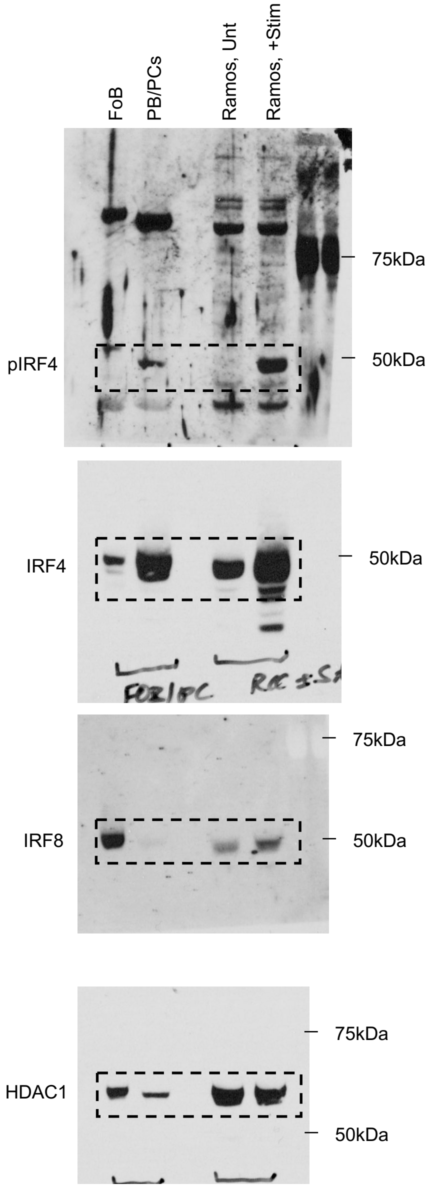

Figure 2A

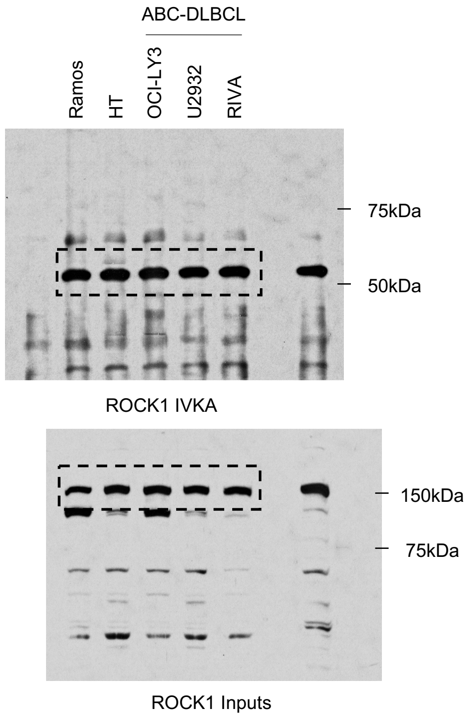

Figure 2B

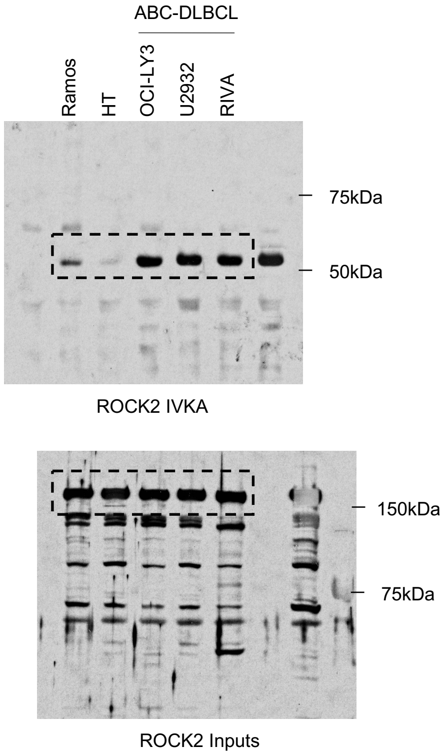

Full Uncut Gels:

Figure 2C

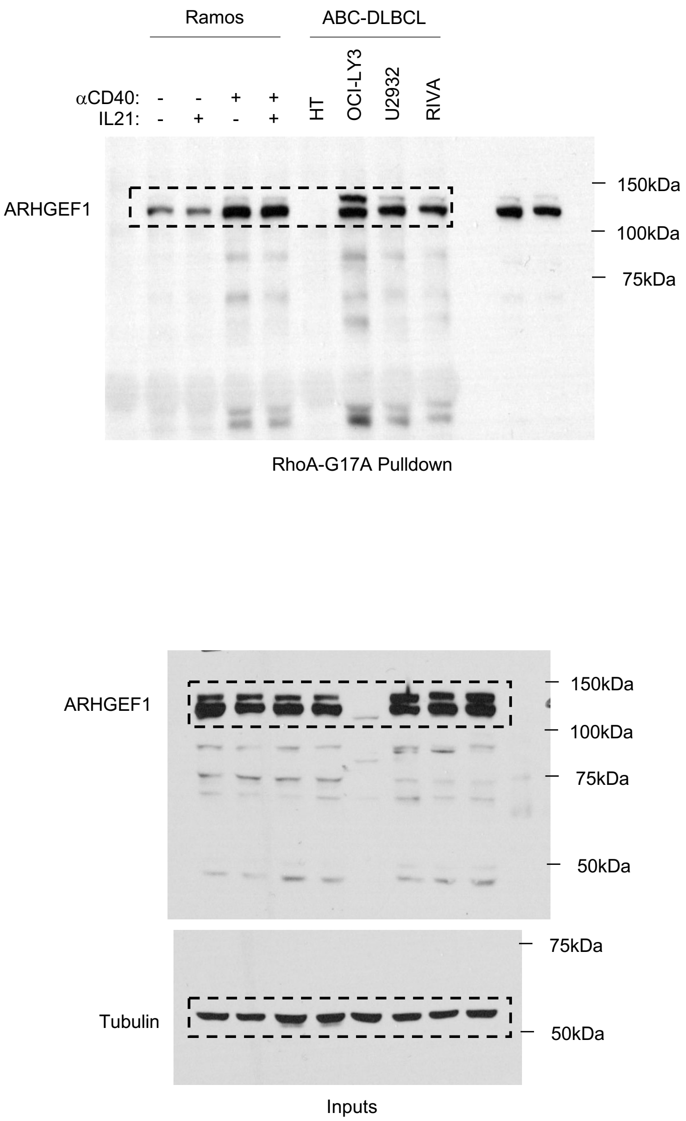

Figure 3A

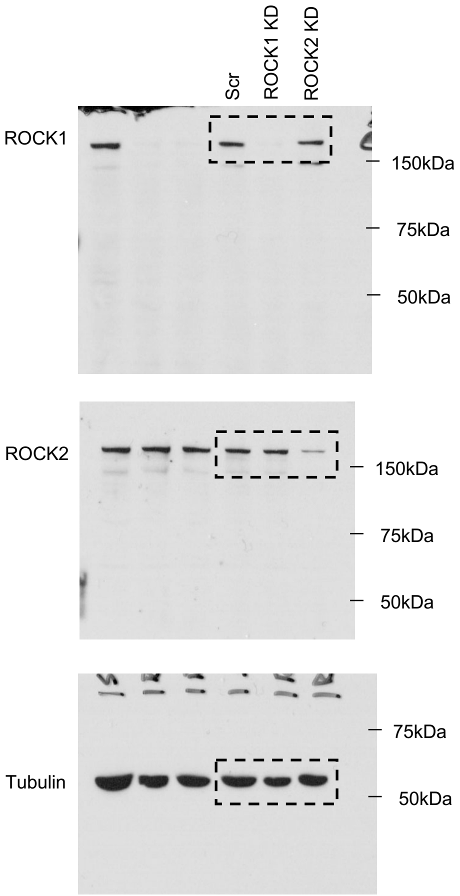

Full Uncut Gels:

Figure 3B

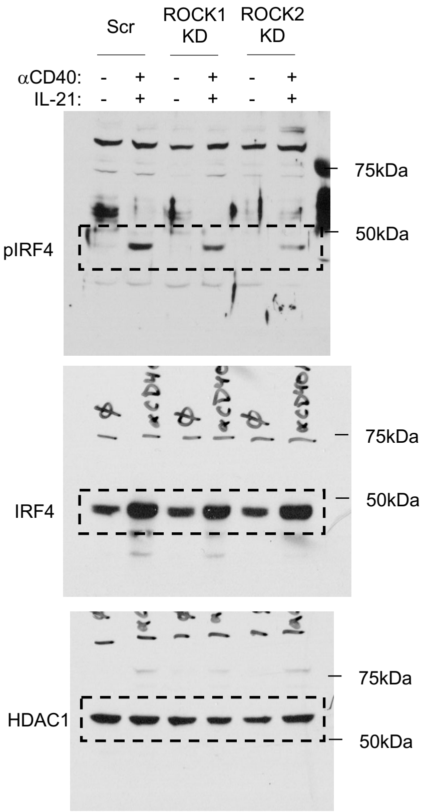

Figure 3G

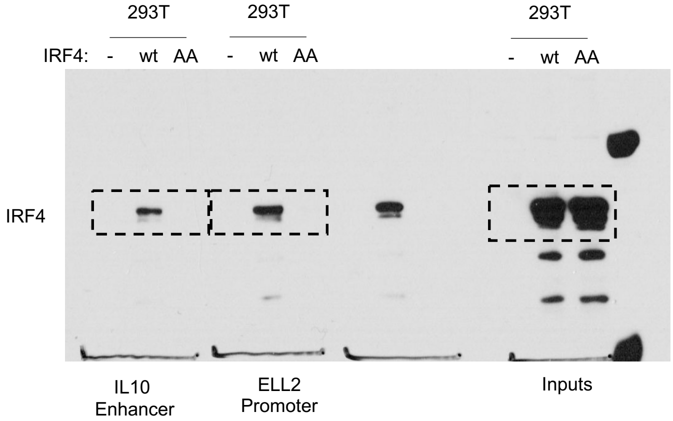

Figure 3H

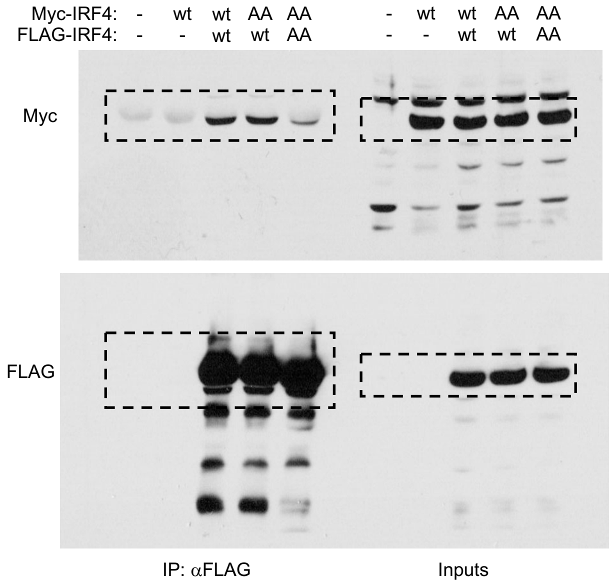

Full Uncut Gels:

Figure 4A

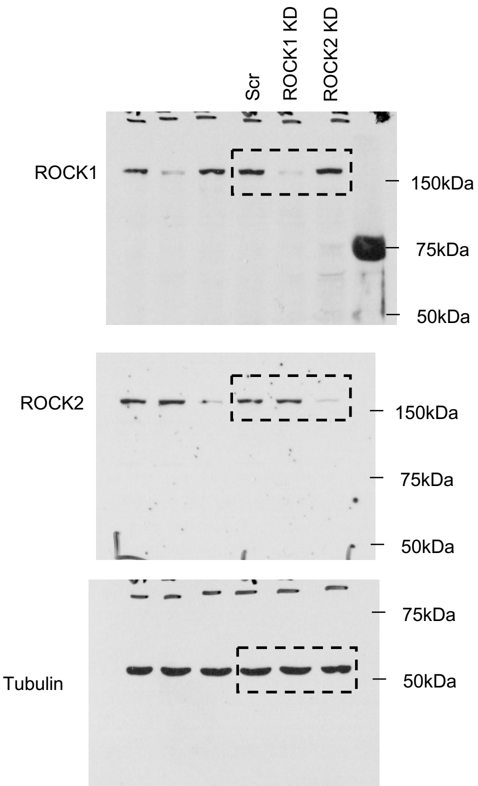

Figure 5A

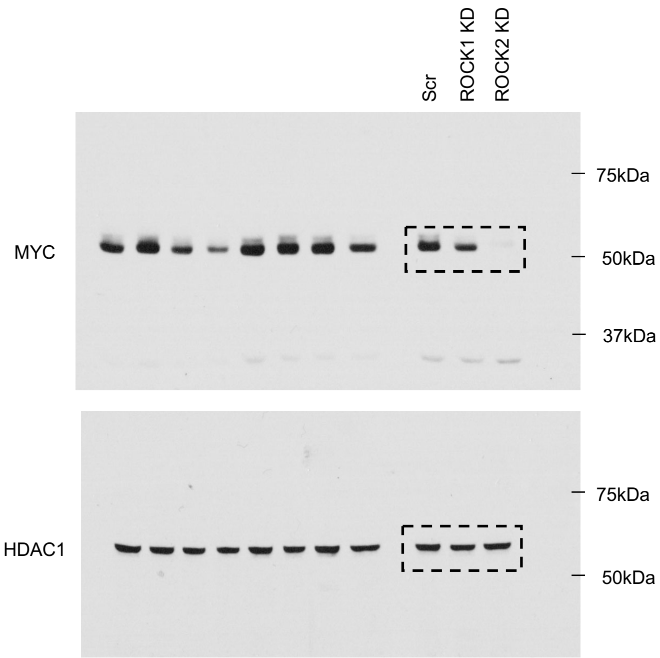

Figure 4B

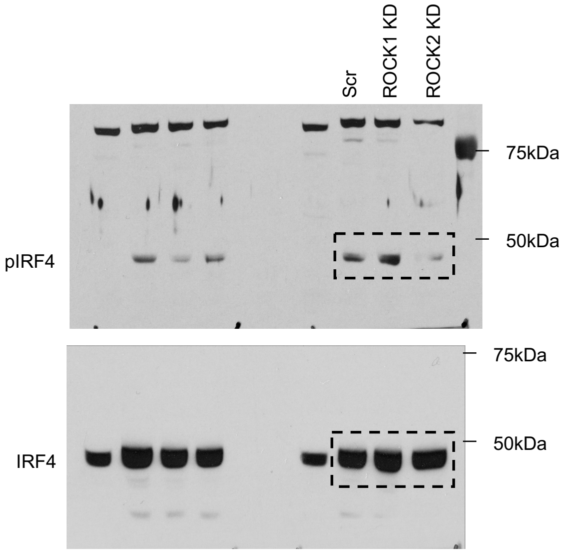

Figure 5D

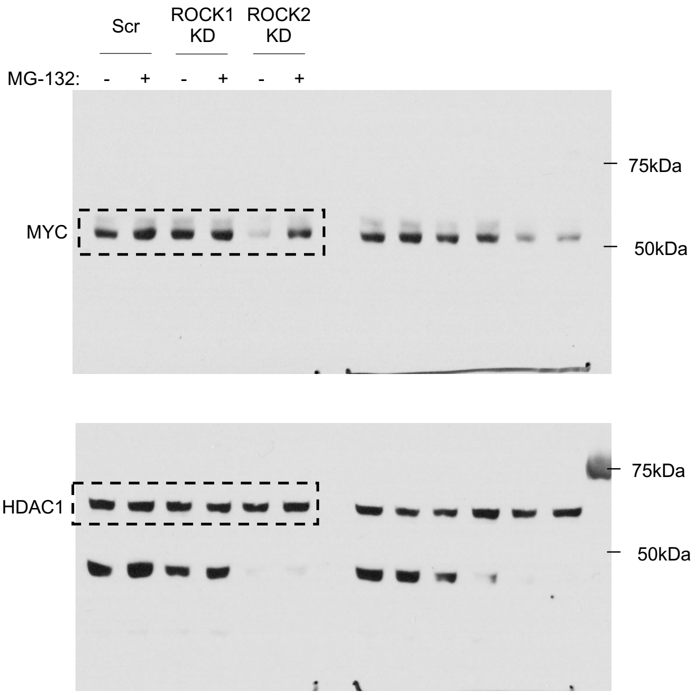

Full Uncut Gels:

Figure 5F

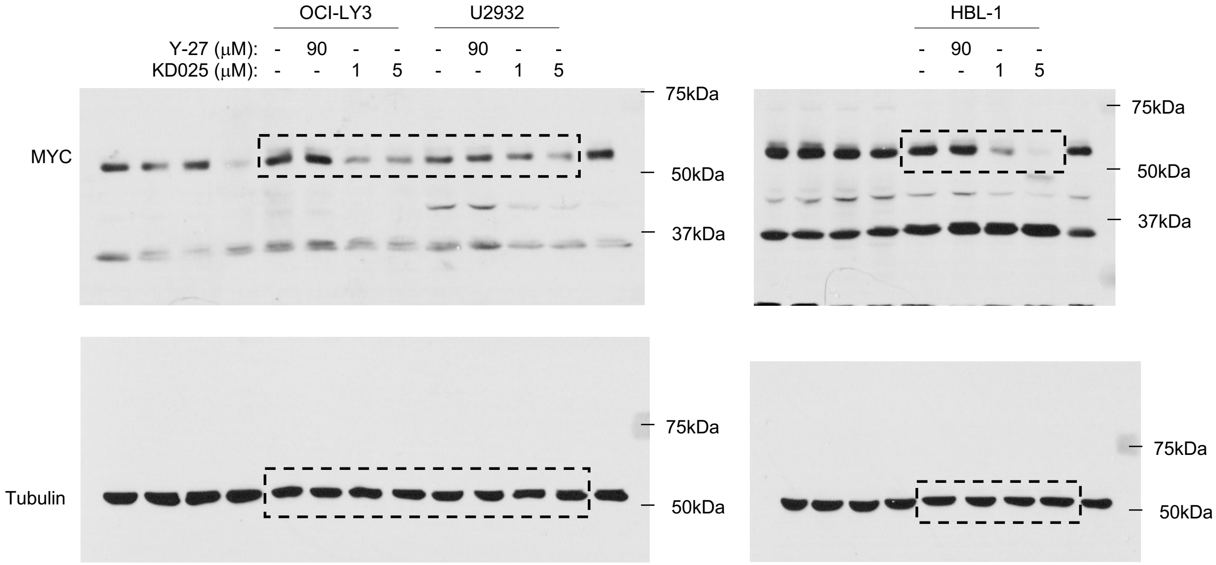

Figure 5G

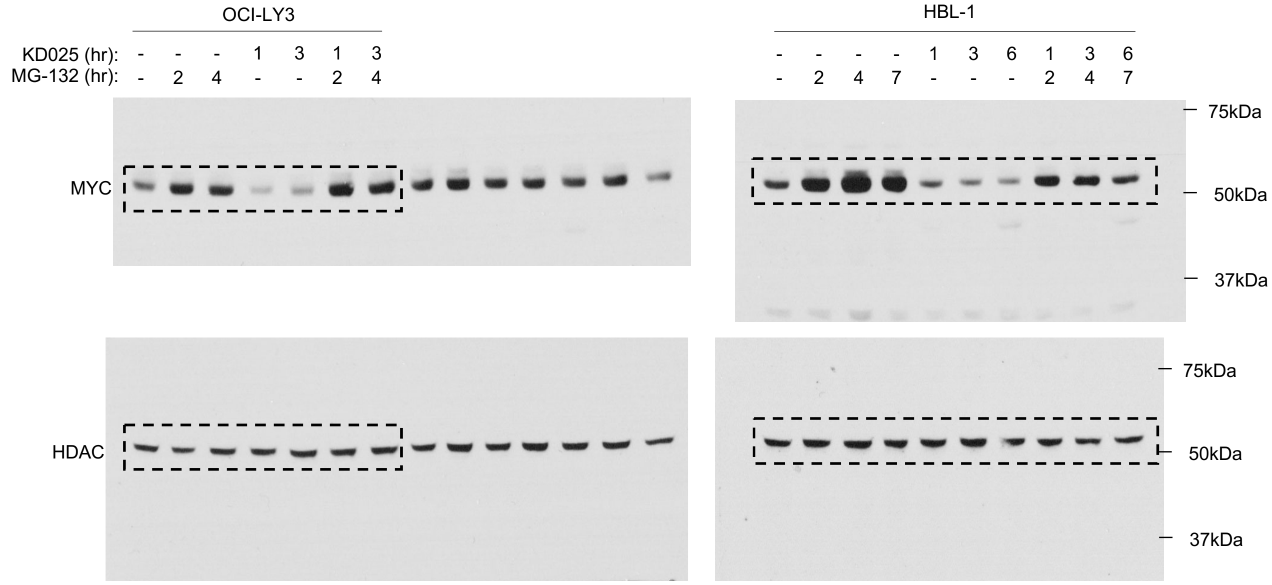

Full Uncut Gels:

Figure S1A

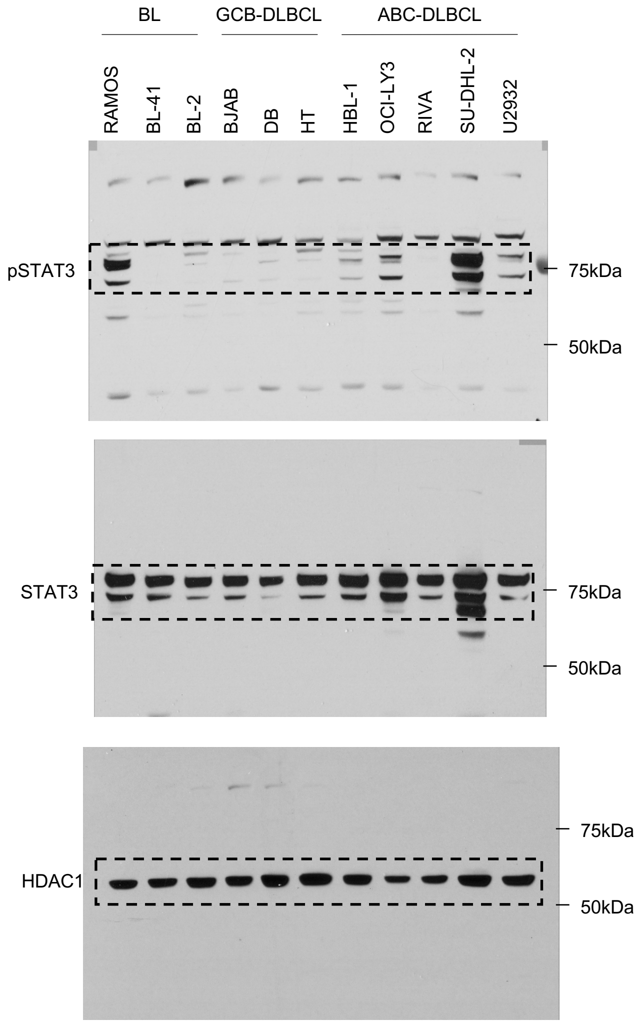

Figure S2B

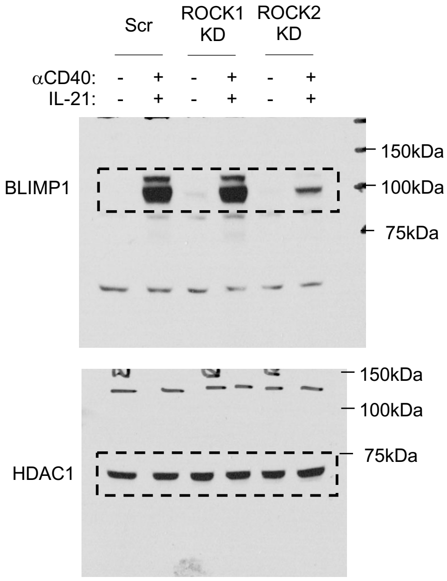

Full Uncut Gels:

Figure S4E

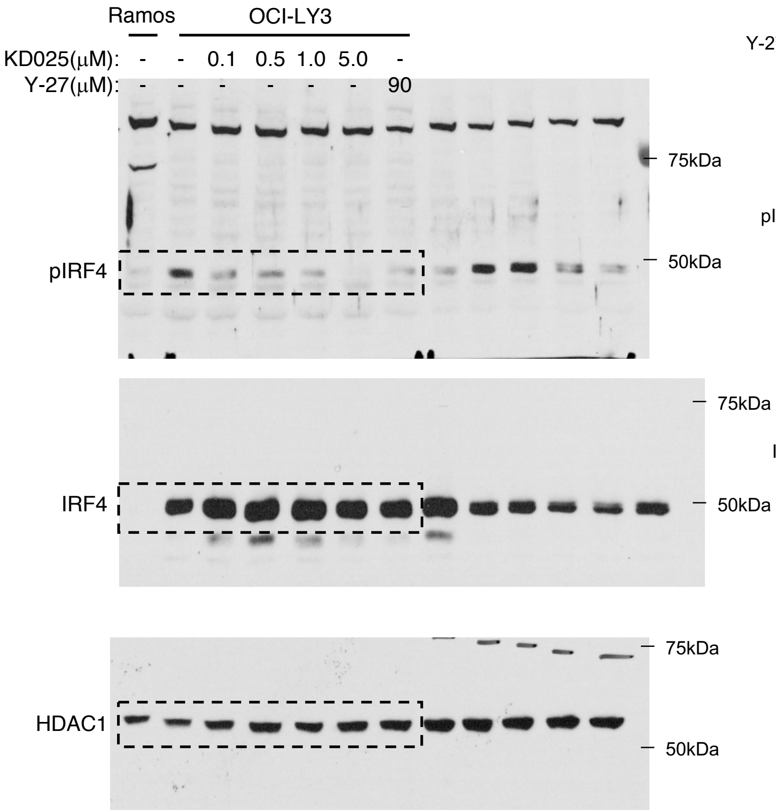

Figure S6B

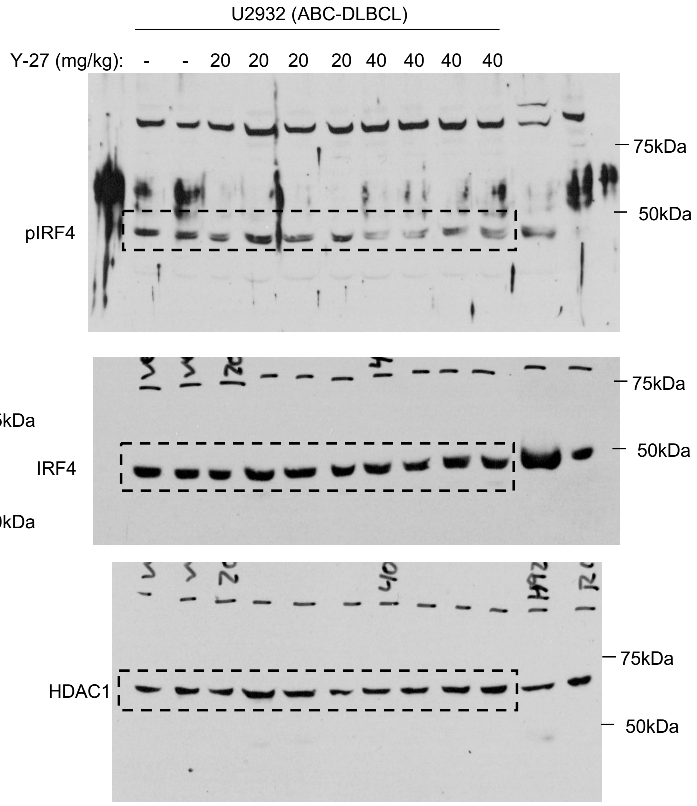

Data for Reviewers

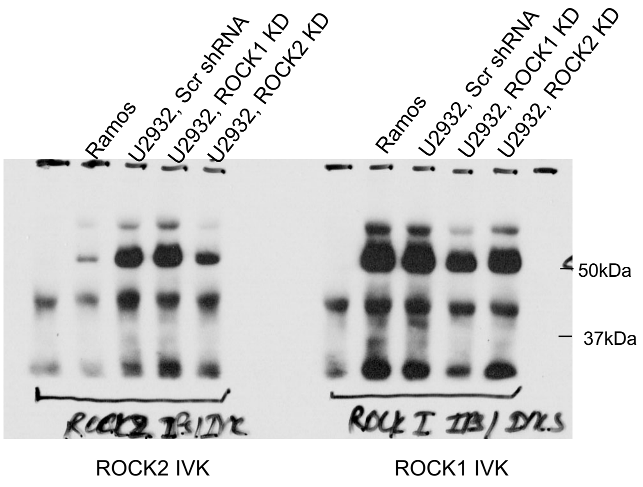

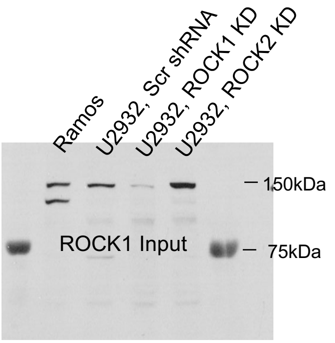

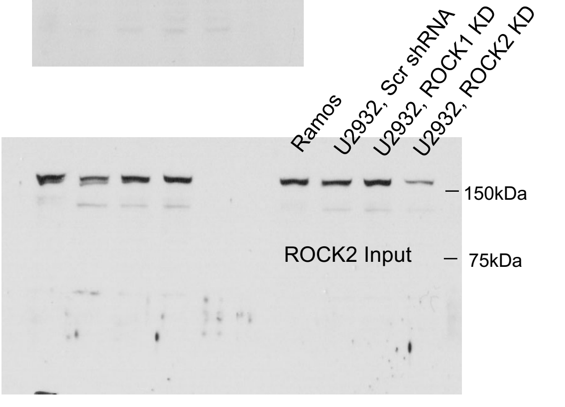

Data for Reviewers.

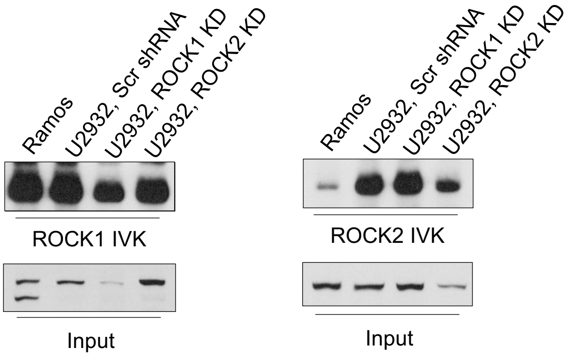

Data for Reviewers. ROCK1 and ROCK2 IVKs in U2932 cells following lentiviral knockdown of ROCK1, ROCK2, or following expression of a non-specific shRNA construct (Scr shRNA).
