# Supplementary material for: Selective dysregulation of ROCK2 activity promotes aberrant transcriptional networks in ABC diffuse large B-cell lymphoma
Source: Sci Rep. 2020 Aug 4;10:13094. doi: 10.1038/s41598-020-69884-1 (PMC7403583; doi:10.1038/s41598-020-69884-1)
Supplement: Supplementary file 6 — Supplementary Table Legends. [file 41598_2020_69884_MOESM6_ESM.docx]

**Supplementary Table S1. Differentially Expressed Genes in U2932 ROCK2 KD versus Scrambled shRNA Controls.** Table showing the logFC of differentially expressed genes (FDR q <0.05) in U2932 following ROCK2 knockdown.

**Supplementary Table S2. Upstream Regulator EnirchR Analysis of Differentially Expressed Genes Between U2932 ROCK2 KD versus Scrambled shRNA Cells.** Table showing the significant upstream regulators from an EnrichR analysis of genes differentially expressed (FDR q <0.05) in U2932 following ROCK2 knockdown.

**Supplementary Table S3. CPDB Pathway Analysis for MYC-Regulated Targets from Upstream Regulator Analysis of ROCK2 KD versus Scrambled shRNA Cells.** Table showing the Consensus Path Database (CPDB) pathway analysis of MYC-regulated targets in the U2932 ROCK2 knockdown geneset as determined in Supplementary Table S2.

**Supplementary Table S4. Oligonucleotides.** List of qPCR primers *(I)*, ChIP-qPCR primers *(II)*, oligonucleotides for ONP assays *(III)*, and lentiviral shRNA sequences *(IV)* used in this study.
